# An endophytic fungus isolated from finger millet (*Eleusine coracana*) produces anti-fungal natural products

**DOI:** 10.3389/fmicb.2015.01157

**Published:** 2015-10-21

**Authors:** Walaa K. Mousa, Adrian Schwan, Jeffrey Davidson, Philip Strange, Huaizhi Liu, Ting Zhou, France-Isabelle Auzanneau, Manish N. Raizada

**Affiliations:** ^1^Department of Plant Agriculture, University of GuelphGuelph, ON, Canada; ^2^Department of Pharmacognosy, Mansoura UniversityMansoura, Egypt; ^3^Department of Chemistry, University of GuelphGuelph, ON, Canada; ^4^Agriculture and Agri-Food Canada, Guelph Food Research CentreGuelph, ON, Canada

**Keywords:** finger millet, *Phoma*, endophyte, *Fusarium*, anti-fungal natural products, viridicatol, tenuazonic acid, alternariol

## Abstract

Finger millet is an ancient African cereal crop, domesticated 7000 years ago in Ethiopia, reaching India at 3000 BC. Finger millet is reported to be resistant to various fungal pathogens including *Fusarium* sp. We hypothesized that finger millet may host beneficial endophytes (plant-colonizing microbes) that contribute to the antifungal activity. Here we report the first isolation of endophyte(s) from finger millet. Five distinct fungal species were isolated from roots and predicted taxonomically based on 18S rDNA sequencing. Extracts from three putative endophytes inhibited growth of *F. graminearum* and three other pathogenic *Fusarium* species. The most potent anti-*Fusarium* strain (WF4, predicted to be a *Phoma* sp.) was confirmed to behave as an endophyte using pathogenicity and confocal microscopy experiments. Bioassay-guided fractionation of the WF4 extract identified four anti-fungal compounds, viridicatol, tenuazonic acid, alternariol, and alternariol monomethyl ether. All the purified compounds caused dramatic breakage of *F. graminearum* hyphae *in vitro*. These compounds have not previously been reported to have anti-*Fusarium* activity. None of the compounds, except for tenuazonic acid, have previously been reported to be produced by *Phoma*. We conclude that the ancient, disease-tolerant crop, finger millet, is a novel source of endophytic anti-fungal natural products. This paper suggests the value of the crops grown by subsistence farmers as sources of endophytes and their natural products. Application of these natural chemicals to solve real world problems will require further validation.

## Introduction

Finger millet [*Eleusine coracana* (L.) Gaertn.] is an important cereal crop widely grown by subsistence farmers in Africa and South Asia (Vietmeyer, 1996; Goron and Raizada, 2015). Finger millet is known in many ancient languages, referred to as *wimbi* (in Swahili), *bule* (Bantu), *dagussa/tokuso* (Amharic), *mugimbi* (Kikuyu), *tailabon* (Arabic), and *ragi* (in South Asian languages) (Vietmeyer, 1996). Finger millet was domesticated in Western Uganda and Ethiopia around 5000 BC then reached India by 3000 BC (Hilu and Wet, 1976). Finger millet is well known by local farmers as a crop that tolerates stress conditions and that resists diverse pathogens (Goron and Raizada, 2015). Though limited scientific research has been conducted on this crop, comparative analysis data from tropical Africa (Burundi) showed that whereas 92–94 identifiable fungal mold species were found in maize grain and 97–99 in sorghum, only 4 were found in finger millet grain (Munimbazi and Bullerman, 1996). Furthermore, whereas 295–327 non-identified mold colonies were found in maize grain, and 508–512 in sorghum, only 4 were found in finger millet grain (Munimbazi and Bullerman, 1996).

The genus *Fusarium* includes widespread pathogens of cereal crops, including *F. verticillioides* in tropical maize which is associated with the production of carcinogenic mycotoxins, and *F. graminearum*, the causal agent of *Gibberella* ear rot in maize and *Fusarium* head blight in wheat; the latter diseases are associated with the mycotoxin deoxynivalenol (DON) (Sutton, 1982). By contrast, in the above African study (Munimbazi and Bullerman, 1996), no *Fusarium* species were identified in finger millet grain, compared to 28–40 *Fusarium* species in maize grain, 11–25 in sorghum, 25–51 in common bean, 4–16 in peanut and 29–43 in mung bean. Other studies, however, have reported the presence of diverse *Fusarium* species in finger millet in India (Pall and Lakhani, 1991; Saleh et al., 2012) and Africa (Amata et al., 2010), including *F. graminearum*, but without apparent plant disease symptoms (Adipala, 1992; Penugonda et al., 2010; Ramana et al., 2012; Saleh et al., 2012). These reports suggest that finger millet may have tolerance to this family of pathogenic fungi—restricting either their presence and/or pathogenicity.

The resistance of finger millet grain to mold has been attributed to abundant polyphenols (Chandrashekar and Satyanarayana, 2006; Siwela et al., 2010). However, an emerging body of literature suggests that microbes that reside in plants without themselves causing disease, defined as endophytes, may contribute to host resistance against fungal pathogens (Johnston-Monje and Raizada, 2011b; Mousa and Raizada, 2013). The mechanisms involved in endophyte-mediated disease resistance include competition for nutrients and space (Haas and Defégo, 2005), induction of host resistance genes (Waller et al., 2005), improvement of host nutrient status (Johnston-Monje and Raizada, 2011a), and/or production of anti-pathogenic natural compounds (Mousa and Raizada, 2013). We hypothesized that endophytes might contribute to the resistance of finger millet to fungal pathogens including members of the genus *Fusarium* as reported by local farmers. *Fusarium* is an ancient fungal genus, dated to at least 8.8 million years ago, and their diversification appears to have co-occurred with that of the C4 grasses (which includes finger millet), certainly pre-dating finger millet domestication in Africa (O'Donnell et al., 2013). A diversity of *F. verticillioides* (synonym *F. moniliforme*) has been observed in finger millet in Africa and it has been suggested that the species evolved there (Saleh et al., 2012). Despite these observations, we could not find reports of endophytes isolated from finger millet.

The objective of this study was to isolate endophytes from finger millet, to investigate endophyte extracts for anti-fungal activity using *F. graminearum*, then to use bio-assay guided fractionation to purify the active anti-*Fusarium* compounds. We report, to the best of our knowledge, the first isolation of endophyte(s) from finger millet. We show that extracts from three putative fungal endophytes have anti-*Fusarium* activity. From the most potent strain, confirmed to be an endophyte, we report the structures of all anti-*Fusarium* compounds, and show the *in vitro* microscopic effects of these compounds against *F. graminearum*.

## Materials and methods

### Isolation of finger millet endophytes

Finger millet seeds were of commercial food grade, originating from Northern India. Plants were grown under semi-hydroponic conditions (i.e., all nutrients provided in solution; on clay Turface MVP, Profile Products, Buffalo Grove, Illinois, USA) in 22.5 L pales placed in the field (Arkell Field Station, Arkell, ON, Canada, GPS: 43°39′ N, 80°25′ W, and 375 m above sea level) during the summer of 2012 and irrigated with the following nutrient solution: urea (46% N content), superphosphate (16% P_2_O_5_), muriate of potash (60% K_2_O), Epsom salt (16% MgO, 13% S), and Plant-Prod Chelated Micronutrient Mix (3 g/L, Plant Products, Catalog #10047, Brampton, Canada) consisting of Fe (2.1 ppm), Mn (0.6 ppm), Zn (0.12 ppm), Cu (0.03 ppm), B (0.39 ppm) and Mo (0.018 ppm). Six tissue pool sets (3 sets of: 5 seeds and 5 root systems from pre-flowering plants) were surface sterilized as follows: samples were washed in 0.1% Triton X-100 detergent for 10 min with shaking; the detergent was decanted, 3% sodium hypochlorite was added (10 min twice for seeds; 20 min twice for roots), followed by rinsing with autoclaved, distilled water, washing with 95% ethanol for 10 min; and finally the samples were washed 5-6 times with autoclaved, distilled water. Effective surface sterilization was ensured by inoculating the last wash on PDA and LB plates at 25°C and 37°C, respectively; all washes showed no growth. Tissues were ground directly in LB liquid medium in a sterilized mortar and pestle, then 50 μl suspensions were plated onto 3 types of agar plates [LB, Potato Dextrose Agar (PDA) and Biolog Universal Growth media (Catalog #70101, Biolog, Inc., Hayward, CA, USA)]. Plates were incubated at 25°C or 37°C for 2–7 days. A total of 10 fungal colonies with unique morphologies were selected and purified by repeated culturing on fresh media.

### Testing the pathogenicity of the isolated endophytes

To test if the most potent putative fungal endophyte identified in this study (WF4) is a pathogen, finger millet seeds were surface sterilized (see above) and planted on sterile Phytagel based medium (7 seeds per tube). The medium was prepared using distilled water as follows (per 2 L): 1 package Murashige and Skoog modified basal salt mixture (Catalog #M571, PhytoTechnology Laboratories, USA), 500 ml nicotinic acid (1 mg/ml), 1 ml pyridoxine HCl (0.5 mg/ml), 10 ml thiamine HCl (100 mg/L), 1 ml glycine (2 mg/ml), 0.332 g CaCl_2_, 1 ml MgSO_4_ (18 g/100 ml), and 4 g Phytagel. The tubes were kept in the dark for 3 days then transferred to light shelves (25°C, 16 h light). When plants were 1 week old, the candidate endophyte (or control) was applied onto the gel surface (as 11 mm diameter agar discs). The negative control consisted of seedlings that received no endophytes, while the positive control consisted of seedlings that received the fungal pathogen *Alternaria alternata*. Disease symptoms were assessed visually at 10 days after inoculation with each endophyte or the controls. There were three replicates (tubes) for each treatment.

### Root colonization study

To test the ability of the putative candidate endophyte (WF4) to colonize the roots of finger millet, confocal microscopy was used. Finger millet seeds were surface sterilized (see above) and planted on sterile Phytagel based medium in glass tubes, each with 4–5 seeds (for medium preparation and growth conditions, see above). The fungal endophyte was applied (as 100 μL of a 48 h old culture grown in potato dextrose broth) to finger millet seedlings (17 days after germination) and incubated at room temperature for 24 h. The control consisted of seedlings incubated with potato dextrose broth only. There were three replicate tubes for the fungal endophyte or the control. Thereafter, roots were stained with Calcofluor (catalog #18909, Sigma Aldrich), following the manufacturer's protocol and scanned with a Leica TCS SP5 confocal laser scanning microscope (Leica Microsystems, Mannheim, Germany) at the Molecular and Cellular Imaging Facility, University of Guelph. The conditions for the scanning were as follows: excitation at 405 nm with an Argon laser (17%) and 488 nm with a green helium laser (25%); the emission ranges were 418–452 nm and 603–689 nm; pinhole [Au] = 1.0 airy; objective lens = 63x oil immersion; and frame average = 3 times.

### Molecular identification of endophytic fungi using 18S rDNA

Genomic DNA from cultured fungal endophytes was extracted using a DNA extraction kit (Fungi/Yeast Genomic DNA Isolation Kit, Catalog #27300, Norgen Biotek Corp, Niagara-on-the-Lake, ON, Canada) and then quantified using a Nanodrop machine (Thermo Scientific, USA). The extracted DNA was used as template to PCR amplify 18S rDNA using universal, degenerate primers as previously described (Borneman and Hartin, 2000). The PCR master mix consisted of the following (per 20 μl): 50 ng/μl DNA, 2.5 μl Standard Taq Buffer (10X, New England Biolabs, USA), 0.5 μl of 25 mM dNTP mix, 1 μl of 10 mM primer nu-SSU-0817-5′ with sequence 5′-TTAGCATGGAATAATRRAATAGGA-3′, 1 μl of 10 mM primer nu-SSU-1536-3′ with sequence ATTGCAATGCYCTATCCCCA, 0.25 μl of 50 mM MgCl_2_, 0.25 μl of Standard Taq (10U/μl, New England Biolabs, USA), and double distilled water up to 20 μl total. PCR amplification conditions were: 96°C for 3 min, followed by 35 amplification cycles (94°C for 30 s, 48°C for 30 s, 72°C for 90 s), and a final extension at 72°C for 7 min, using a PTC200 DNA Thermal Cycler (MJ Scientific, USA). The PCR products were separated on a 1.5% agarose gel at ≤ 5 V/cm; 700 bp sized bands were excised and eluted using a gel purification kit (Illustra GFX 96 PCR Purification kit, GE Healthcare, USA). The purified DNA was sequenced at the University of Guelph Genomic Facility. Five distinct fungal strains were identified based on 18S rDNA sequence comparisons using BLAST searches to Genbank (default parameters for BLASTN: Expect threshold 10, Match/Mismatch Scores 1,−2, Gap Costs: Linear).

### Fermentation of fungal endophytes and antifungal screening

To enable endophytic fungi to grow and secrete metabolites, for each endophyte, 50 g of commercial white rice (variety: Basmati long grain) was placed in 100 ml H_2_0 in a 2 L flask, which was incubated overnight at room temperature, autoclaved, then inoculated with 5 ml of endophytic fungi inoculum; for each inoculum, mycelia were inoculated into potato dextrose broth medium and placed on a shaker at 100 rpm for ~3 days. Endophytic fungi were fermented on rice solid medium for 4 weeks in darkness at room temperature without shaking. Each endophytic fungus was fermented in triplicate. For metabolite extraction: Ethyl acetate was added (3 × 1.5 L) and the samples were incubated overnight at room temperature, filtered [0.2 micron filter paper (Whatman, USA), cotton and/or cheesecloth], then concentrated on a rotary evaporator at 45°C. The ethyl acetate extract was then washed with water to remove salts and sugars. The ethyl acetate fraction was then dried and subject to liquid fractionation between Methanol and *n*-Hexane to remove long chain hydrocarbons. Methanol condensates were stored at 4°C and were used to screen for anti-fungal activity (at a concentration range of 6.8–7.4 mg/ml) and to purify candidate anti-fungal compounds. Methanol condensates were diluted in a minimal amount of methanol and used to screen endophytic fungi for inhibition of growth of *Fusarium graminearum* (obtained from Agriculture and Agrifood Canada, Guelph, ON) using the agar diffusion method: Briefly, *F. graminearum* was grown for 24–48 h (25°C, 100 rpm) in potato dextrose broth medium, then mycelia were added to melted, cooled PDA medium (1 ml of fungus into 100 ml of medium), mixed and poured into Petri dishes (100 × 15 mm), then allowed to re-solidify. Wells (11 mm diameter) were created in this pathogen-embedded agar by puncturing with sterile glass tubes, into which the methanol endophyte extracts were applied (200 μl per well). The agar plates were incubated at 30°C for 48 h in darkness. The radius of each zone of inhibition was measured. The fungicides Amphotericin B (Catalog #A2942, Sigma Aldrich, USA) and Nystatin (Catalog #N6261, Sigma Aldrich, USA) were used as positive controls, and methanol was used as a negative control. Each endophyte was screened in three independent replicates.

### Anti-fungal target spectrum of extracts

Endophyte methanol extracts which tested positive for activity against *F. graminearum* were re-screened for activity against a diversity of other fungi (obtained from Dr. Ting Zhou, Agriculture and Agrifood Canada, Guelph, Canada) using the agar diffusion method (described above) to characterize the activity spectrum of each extract. The fungi tested included: *Alternaria alternata, Alternaria arborescens, Aspergillus flavus, Aspergillus niger, Bionectria ochroleuca, Davidiella* (*Cladosporium*) *tassiana, Diplodia pinea, Diplodia seriata, Epicoccum nigrum, Fusarium lateritium, Fusarium sporotrichioides, Fusarium avenaceum* (*Gibberella avenacea*, two isolates), *Nigrospora oryzae, Nigrospora sphaerica, Paraconiothyrium brasiliense, Penicillium camemberti, Penicillium commune, Penicillium expansum, Penicillium afellutanum, Penicillium olsonii, Rosellinia corticium, Torrubiella confragosa, Trichoderma hamatum*, and *Trichoderma longibrachiatum*.

### General procedure to purify the active anti-*F. graminearum* compounds from WF4

To permit large scale purification of the active compound(s) from the most potent anti-fungal endophyte extract (from *Phoma* endophyte WF4), the fermentation procedure described above was scaled up to five 2 L flasks, each containing 50 g of commercial white rice (variety: Basmati long grain) or millet (Variety: Pearl millet) placed in 100 ml H_2_0. To purify the active compound from WF4, the dry methanol residues were separated by flash liquid chromatography and preparative high pressure liquid chromatography. For flash liquid chromatography, the column was filled with flash grade silica gel (SiliaFlash® P60 230–400, catalog # R12030B, SiliCycle, USA) and saturated with the desired mobile phase just prior to sample loading. Three grams of the dry residue extract were dissolved in chloroform and after rotary evaporation, applied as a dried silica band on the top of the column. The mobile phase (step-wise elution using hexane-ethyl acetate then ethyl acetate-methanol mixture with increasing polarity) was then passed through the column with air pressure resulting in sample separation. For Preparative HPLC, the most appropriate solvent system was determined using analytical HPLC before running the HPLC separation (Waters prep LC 4000 system, USA). The mobile phase combination was purified water (Nanopure, USA) and acetonitrile, pumped in a gradient manner (starting at 99:1 and ends at 0:100). Each injection consisted of 250 mg of the fraction dissolved in 8 ml of the solvent system. The mobile phase was pumped through the column (Nova-Pak HR C18 Prep Column, 6 μm, 60 Å, 25 × 100 mm prepack cartridge, part # WAT038510, Serial No 0042143081sp, Waters Ltd, USA) at a rate of 5 ml/min. The eluted peaks were detected by the UV detector (2487 Dual λ Absorbance Detector, Waters Ltd, USA) and collected separately. Samples were then dried under inert nitrogen gas. Thin layer chromatography (TLC) was used to visualize the bands using TLC glass silica gel (60 F254, Whatman, Germany). Chromatograms were developed using different solvents and detected under UV light (254 and 366 nm), and heated at 95°C after spraying with vanillin-H_2_SO_4_ reagent (0.5 g vanillin dissolved in a mixture of 85 ml methanol, 10 ml acetic acid and 5 mL H_2_SO_4_). The fractions were dried by rotary evaporator, re-dissolved in their solvents and screened for antifungal activity as described above. Purified fractions were screened in the anti-fungal disc diffusion assay (against *Fusarium graminearum*, described above) to identify the active fraction(s). The active fractions were further purified. The active anti-fungal compounds (20 μl of 5mg/ml) were rescreened against *F. graminearum* using the agar disc diffusion method, and then subjected to structural analysis as described below.

### Structure elucidation of the anti-fungal compounds from WF4

The chemical structures of the anti-fungal compounds isolated from WF4 were elucidated primarily using one and two dimensional nuclear magnetic resonance (NMR) techniques in combination with mass spectrometry (MS) methods. Further spectroscopic methods, such as IR (infra-red) spectroscopy provided additional structural information. All NMR spectra were conducted at the Guelph NMR Facility using a 600 DPX spectrometer (Bruker, Germany) operating at 600 MHz. The spectra were recorded in CDCl_3_ and DMSO-*d6*. Structural assignments were based on spectra resulting from the following NMR experiments: ^1^H, APT, ^1^H-^1^H COZY, ^1^H-^13^C direct correlation (HSQC), ^1^H-^13^C long range correlation (HMBC). IR spectroscopy was conducted using a Bruker Alpha IR Spectrometer instrument (Bruker, Germany) located in the Department of Chemistry, University of Guelph. MS was conducted in the mass spectroscopy facility of the Advanced Analysis Centre at the University of Guelph using the following acquisition parameters; Ion Source (ESI), Ion Polarity (Positive), Alternating Ion Polarity (off), capillary exit (Resolution, 140.0 V), Mass Range Mode (Enhanced), Scan Begin (70 m/z), Scan End (1000 m/z), Trap Drive (58.9), Accumulation Time (1348 μs), Averages (3 Spectra), Auto MS/MS (on).

### Microscopic mechanisms of action

Microscope slides were used to view the *in vitro* interactions between pathogen and each purified compound. Each slide was coated with a thin layer of PDA, then 20 μl of each purified compound (5 mg/ml) was applied adjacent to 100 μl of *F. graminearum* (mycelia grown for 24–48 h in potato dextrose broth at 25°C, with shaking at 100 rpm). For the positive control, 20 μl of PROLINE® 480 SC fungicide (Bayer CropScience, USA) was used (at a concentration of 48 g/L of the active ingredient Prothioconazole). Each slide was incubated at 25°C for 24 h then stained with neutral red (Sigma Aldrich, Catalog #57993) or Evans blue (Sigma Aldrich, Catalog # E2129) by placing 100 μl of stain on the slide, followed by a 3–5 min incubation, then washing 3–4 times with deionized water. Pictures were taken using a light microscope (MZ8, Leica, Wetzlar, Germany for neutral red staining; and BX51, Olympus, Tokyo, Japan for Evans blue staining). There were 3–4 replicates for each slide.

### Statistical analysis

All statistical analysis was performed using Prism Software version 5 (Graphpad Software, USA). All error bars shown represent the range of data points.

## Results

### Isolation and identification of fungal endophytes from finger millet

**Figure 1:**
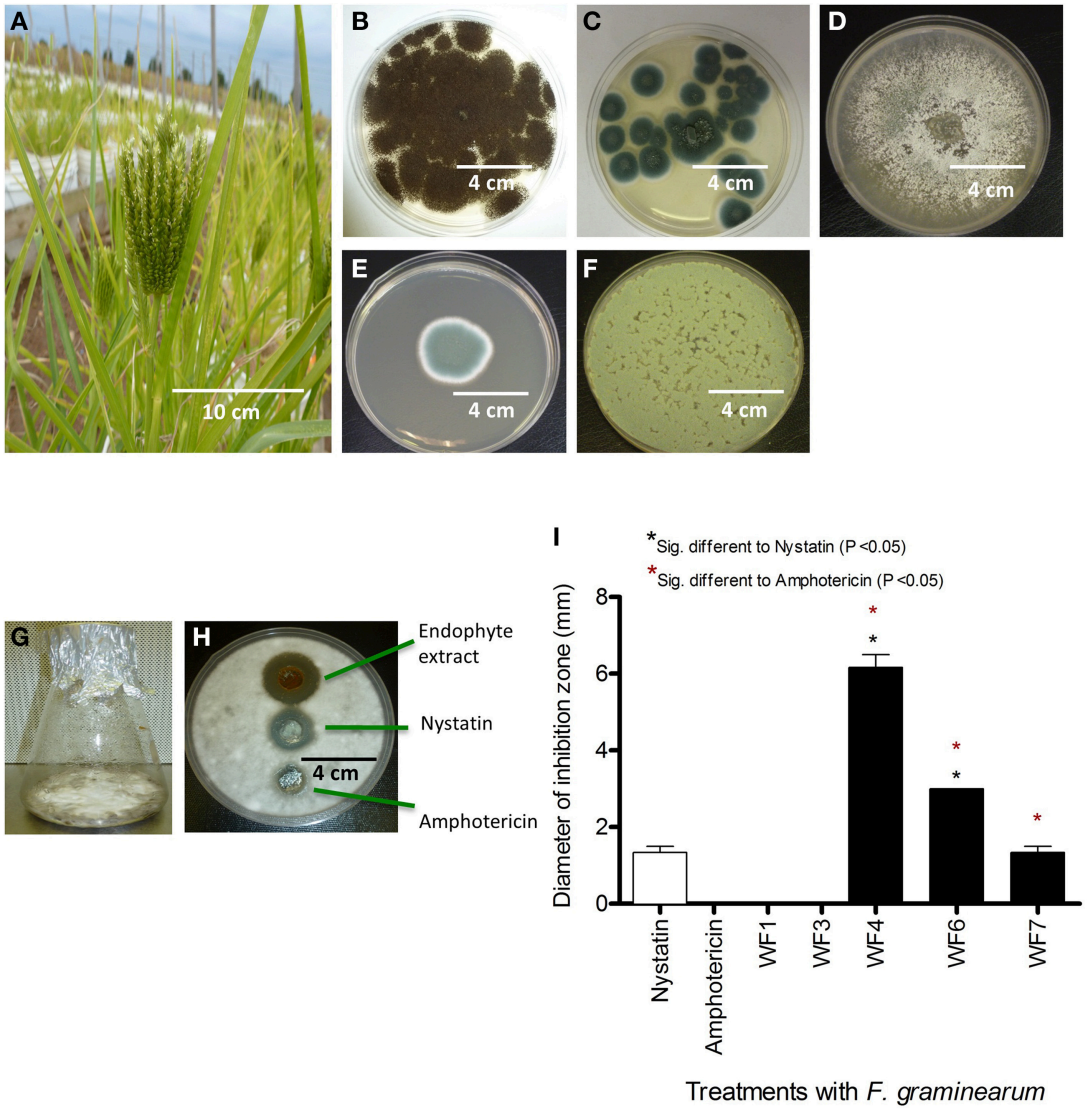
**Isolation of fungal endophytes from finger millet and screen for anti-***Fusarium*** activity from endophyte extracts**. **(A)** Picture of a finger millet plant. **(B–F)** Pictures of fungal endophytes isolated from finger millet roots, with the corresponding best BLAST match taxonomic identification in brackets based on 18S rDNA sequencing: **(B)** WF1 (*Aspergillus niger*), **(C)** WF3 (*Penicillium griseofulvum*), **(D)** WF4 (*Phoma* sp.), **(E)** WF6 (*Penicillium chrysogenum*), **(F)** WF7 (*Penicillium expansum*). **(G)** An example of an endophyte fermented on rice medium. **(H)** Example of an endophyte extract showing suppression of *F. graminearum* hyphae (white) using the agar diffusion method, along with fungicide controls. **(I)** Quantification of the effects of different endophyte extracts on the growth of *F. graminearum* (diameter of inhibition zone, *n* = 3).

To isolate fungal endophytes from finger millet, non-sterilized seeds were germinated and grown in non-sterilized clay Turface in pails placed in the field (Figure 1A). Three replicate pools of seeds and intact root systems, each consisting of five samples (plants at pre-flowering stage), were surface sterilized. Sterilized tissues were ground in LB liquid medium and the extracts were plated onto LB agar, Potato Dextrose Agar (PDA) and Biolog Universal Growth media. A total of 10 fungal strains with diverse morphologies were isolated from roots; no fungal endophytes were isolated from seeds. 18S rDNA sequencing and BLAST searches predicted only five distinct fungal strains, which were named WF1 (GenBank # KF957636), WF3 (GenBank # KF957638), WF4 (GenBank # KF957639), WF6 (GenBank # KF957641), and WF7 (GenBank # KF957642) (Supplemental Tables S1, S2, Figures 1B–F). The fungal endophytes of finger millet most closely resembled the genera *Aspergillus, Penicillium* and *Phoma* based on 18S rDNA sequence identities (Supplemental Tables S1, S2).

### *In vitro* screen for endophyte extracts with anti-fungal activity

Solid rice culture medium was used to ferment the putative endophytic fungi (Figure 1G), then their concentrated methanol extracts were used to test for activity against *F. graminearum* using the agar diffusion method (Figure 1H). Briefly, *F. graminearum* was embedded in agar; endophyte extracts were applied in parallel to standard fungicide controls (Nystatin, Amphotericin), and then any resulting zones of inhibition of *F. graminearum* growth were measured. Extracts from three of the five putative endophytes (WF4, WF6, WF7) consistently inhibited growth of *F. graminearum*, with the extract from WF4 causing the greatest inhibition, and WF7 causing the least inhibition (Figure 1I).

To determine the target spectrum of anti-fungal activity, the three positive extracts were then tested against 25 genetically diverse fungal strains (Table 1). Extracts from WF4- WF6 and WF7 were observed to inhibit the growth of six of the 25 fungi, of which three were pathogenic strains of *Fusarium* (*Fusarium lateritium, Fusarium sporotrichioides, Fusarium avenaceum*); these extracts also inhibited two other known fungal crop pathogens (*Alternaria alternata, Aspergillus flavus)* in addition to the soil fungus, *Trichoderma longibrachiatum* (Table 1). None of the endophytic extracts were observed to be specifically targeted to *Fusarium* species. The extract from WF4 was observed to have the widest target spectrum (7/26 fungal strains) and caused the greatest inhibition zone in all cases (Table 1).

**Table 1:** **Effect of endophyte extracts on the growth of a diversity of fungal species**.

**Target fungal species**	Mean diameter^*^ of inhibition zone with each extract (mm)				
	**WF4**	**WF6**	**WF7**	**Nystatin (10 μg/ml)**	**Amphotericin B (5 μg/ml)**
*Alternaria alternata*	3.0 ± 0.2	0.0 ± 0.0	0.0 ± 0.0	0.0 ± 0.0	0.0 ± 0.0
*Alternaria arborescens*	0.0	0.0	0.0	0.0	0.0
*Aspergillus flavus*	4.0 ± 0.2	3 ± 0.2	3.0 ± 0.0	0.0 ± 0.0	1.0 ± 0.2
*Aspergillus niger*	0.0	0.0	0.0	0.0	0.0
*Bionectria ochroleuca*	0.0	0.0	0.0	0.0	0.0
*Davidiella tassiana*	0.0	0.0	0.0	0.0	0.0
*Diplodia pinea*	0.0	0.0	0.0	0.0	0.0
*Diplodia seriata*	0.0	0.0	0.0	0.0	0.0
*Epicoccum nigrum*	0.0	0.0	0.0	0.0	0.0
*Fusarium avenaceum—Isolate 1*	0.0	0.0	0.0	0.0	0.0
*Fusarium graminearum*	6.5 ± 0.2	3.0 ± 0.0	1.5 ± 0.2	1.5 ± 0.2	0.0 ± 0.0
*Fusarium lateritium*	6.0 ± 0.5	0.0 ± 0.0	2.5 ± 0.5	0.0 ± 0.0	1.0 ± 0.2
*Fusarium sporotrichioides*	4.0 ± 0.2	2.0 ± 0.2	3.0 ± 0.2	1.0 ± 0.2	1.0 ± 0.0
*Fusarium avenaceum—Isolate 2 (Gibberella avenacea)*	4.5 ± 0.3	3.5 ± 0.3	0.0 ± 0.0	0.0 ± 0.0	0.0 ± 0.0
*Nigrospora oryzae*	0.0	0.0	0.0	0.0	0.0
*Nigrospora sphaerica*	0.0	0.0	0.0	0.0	0.0
*Paraconiothyrium brasiliense*	0.0	0.0	0.0	0.0	0.0
*Penicillium afellutanum*	0.0	0.0	0.0	3.0 ± 0.2	3.0 ± 0.3
*Penicillium camemberti*	0.0	0.0	0.0	2.0 ± 0.3	5.0 ± 0.3
*Penicillium commune*	0.0	0.0	0.0	1.5 ± 0.2	3.5 ± 0.2
*Penicillium expansum*	0.0	0.0	0.0	2.0 ± 0.2	4.5 ± 0.3
*Penicillium olsonii*	0.0	0.0	0.0	0.0	0.0
*Rosellinia corticium*	0.0	0.0	0.0	0.0	0.0
*Torrubiella confragosa*	0.0	0.0	0.0	0.0	0.0
*Trichoderma hamatum*	0.0	0.0	0.0	0.0	0.0
*Trichoderma longibrachiatum*	8.5 ± 0.3	5.5 ± 0.2	7.0 ± 0.5	2.0 ± 0.2	3.5 ± 0.3

* *Data expressed as mean ± standard error of the mean (SEM), n = 3 or 4 replicates*.

### Toxicity assays of the WF4 extract

The general toxicity of the WF4 extract was then tested using three standard assays. First, maize leaf punches were immersed in different dilutions of each endophyte extract, which were then incubated in the dark (Figure S3A); the WF4 extract showed toxicity against maize leaf punches as indicated by lesions (Figure S3B). In the second assay, the effect of the endophyte extract on the germination of maize and wheat seeds was tested (Figures S3C–F); the extract did not significantly inhibit germination. Finally, the effect of the endophyte extracts on the development of fruit flies (*Drosophila melanogaster*) was monitored from egg hatching to larval instar stages, to encapsulation into pupa, and finally metamorphosis into adult flies (Figure S3G). The undiluted endophyte extract (or buffer control) was applied to six sets of eggs (each set = 30 eggs) and the onset and duration of each developmental transition was monitored over 23 days. The WF4 extract significantly inhibited egg hatching, did not delay the subsequent encapsulation of larva into pupa, but dramatically delayed the metamorphosis from pupa to adult flies (Figure S3H); however, the timing when each developmental stage peaked was significantly delayed and diminished compared to the control (*p* < 0.05, Mann Whitney). Taking into account both the leaf and insect assays, we conclude that the WF4 extract shows toxicity against plants and insects.

### Confirming the endophytic nature of WF4

An endophyte is defined as a microbe that is able to colonize the internal tissues of its plant host without causing disease (Wilson, 1995). To test these criteria, two experiments were undertaken for WF4, the focus of this study:

#### Pathogenicity

**Figure 2:**
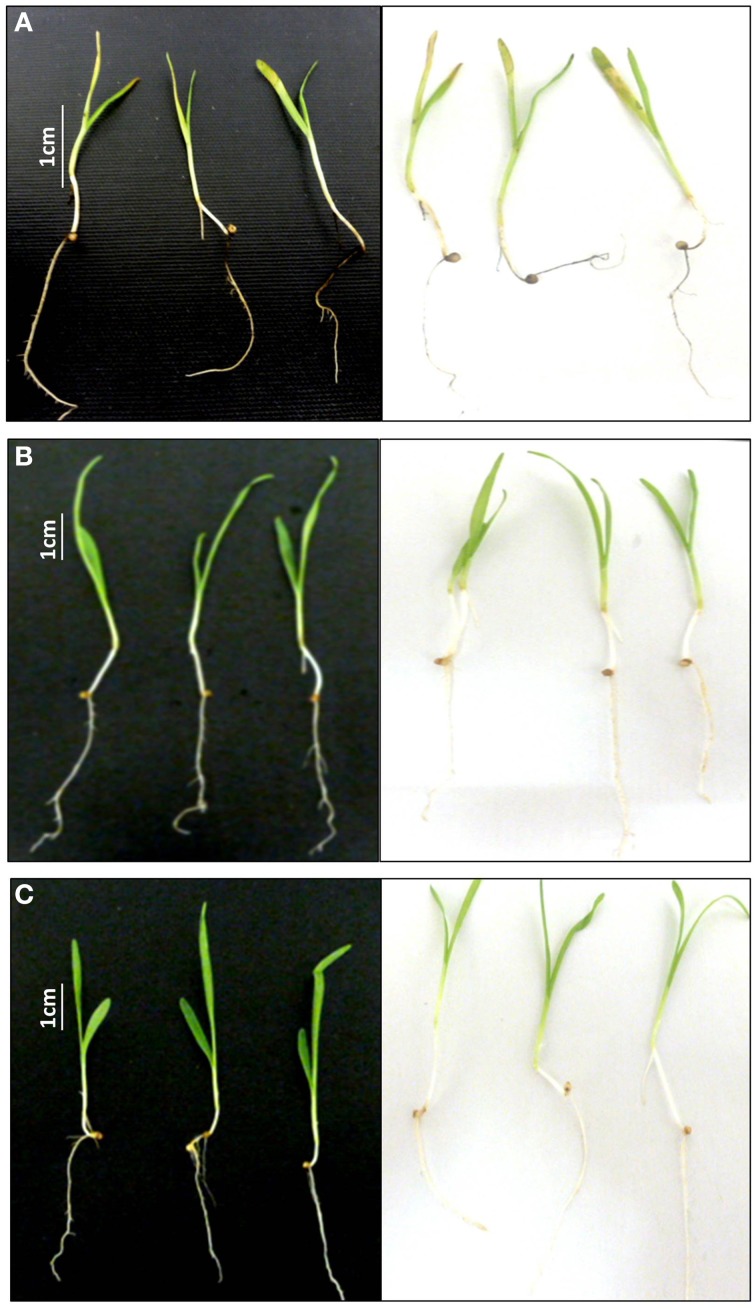
**Testing the pathogenicity of the putative endophyte WF4 on finger millet**. **(A)** Representative pictures showing the effect of the known pathogen *Alternaria alternata* on finger millet seedlings (negative control). **(B)** Representative pictures showing the effect of buffer on finger millet seedlings (positive control). **(C)** Representative pictures showing the effect of the fungus WF4 on finger millet seedlings.

Finger millet seedlings were incubated with WF4 or with *Alternaria alternata*, a known pathogen of finger millet (Kumar, 2010). The seedlings incubated with *A. alternata* developed characteristic disease symptoms including reduction in plant length, black roots and leaf spots (Figure 2A), compared to the healthy buffer control (Figure 2B). In comparison, there were no disease symptoms observed in the seedlings challenged with WF4 (Figure 2C).

#### Plant colonization

**Figure 3:**
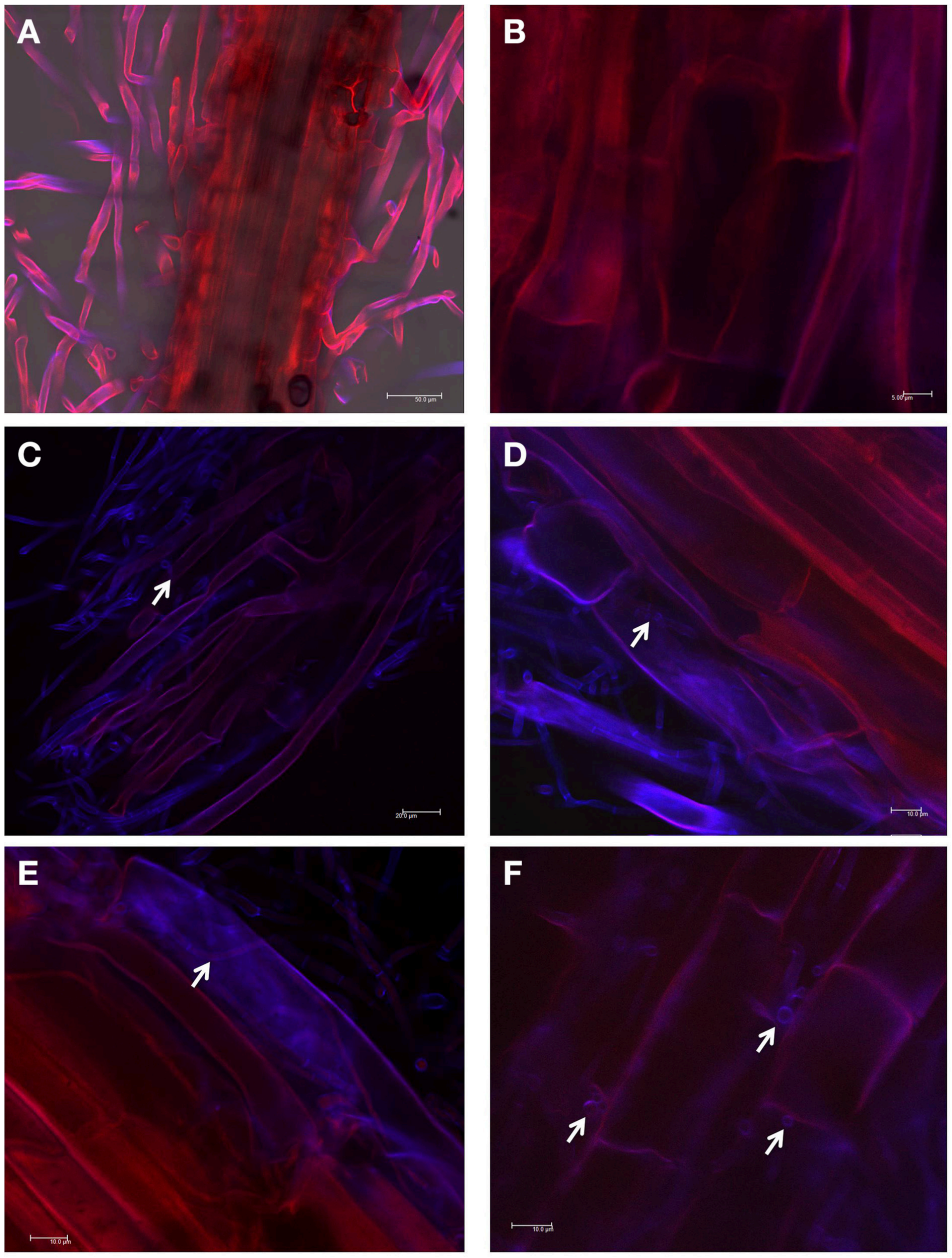
**Root colonization study using confocal scanning laser microscopy**. **(A,B)** Representative pictures of root tissues inoculated with the buffer control. **(C–F)** Representative pictures of root tissues inoculated with WF4. WF4 fluoresces purple-blue due to staining with Calcofluor. Plant tissues appear red due to autofluorescence. White arrows point to WF4 inside the plant tissues.

To test the ability of WF4 to colonize the roots of finger millet (from where it was originally isolated), confocal imaging was used. Seedlings that were inoculated with the buffer only (control) showed no observable fungal growth inside the tissues (Figures 3A,B). In comparison, WF4 could efficiently colonize the root tissues including the epidermis and sub-epidermal layers (Figures 3C–F).

### Bio-assay guided purification and structure elucidation of the anti-fungal compounds from endophyte WF4

**Figure 4:**
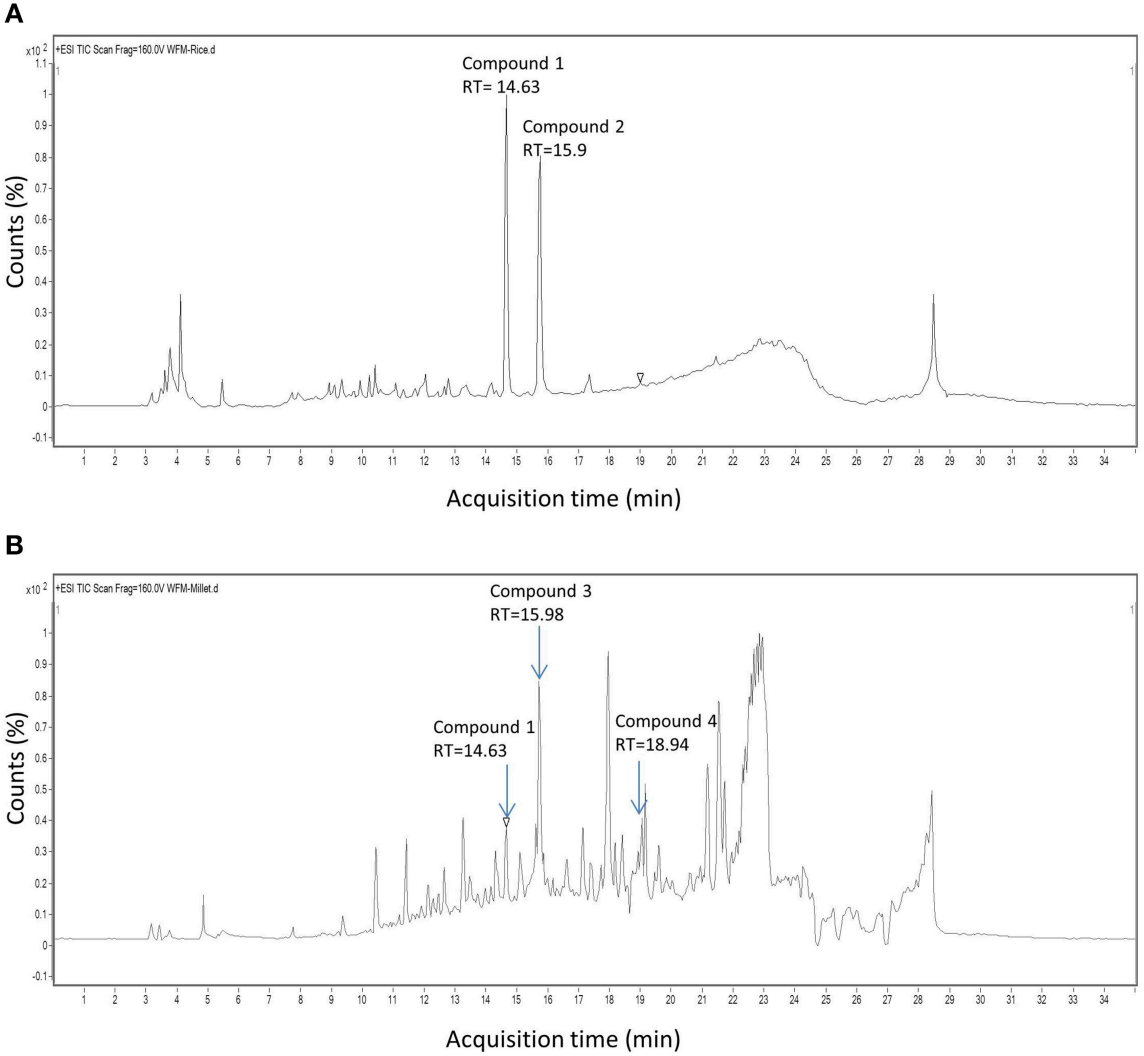
**HPLC chromatograms of extracts derived from endophyte WF4**. Shown are chromatograms when the endophyte was cultured on **(A)** rice medium and **(B)** millet medium. The arrows show the retention time of peaks exhibiting antifungal activity.

**Figure 5:**
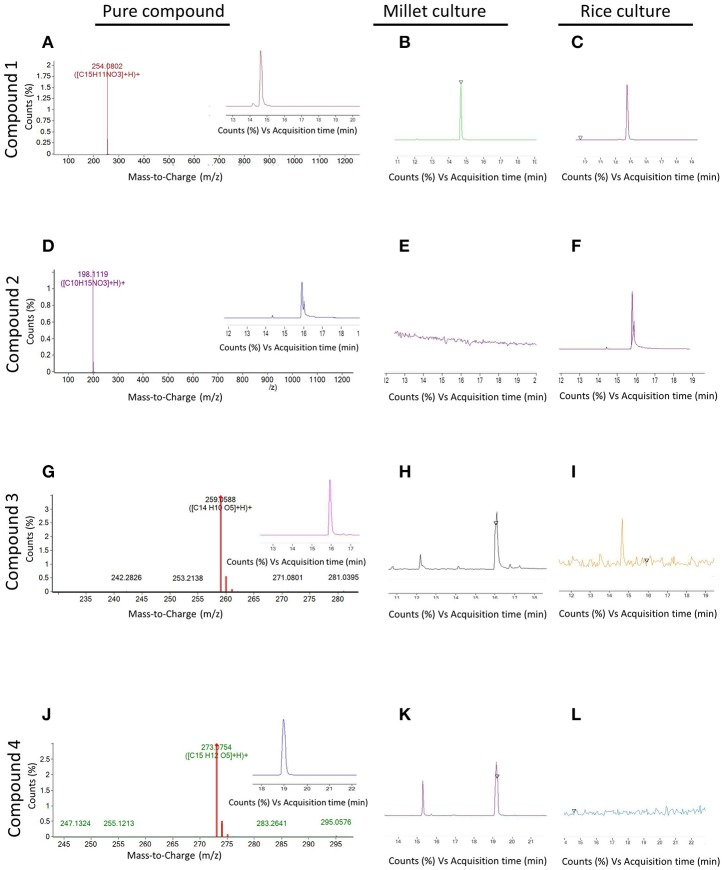
**Mass spectroscopy of peaks with antifungal activity detected in extracts from endophyte WF4**. **(A,D,G,J)** (M+H^+^) values of compounds 1, 2, 3, and 4 respectively. The insets show the retention time of each respective compound. **(B,E,H,K)** Magnification within the whole spectrum of the extract of WF4 cultured on millet medium, showing the absence or presence of peaks corresponding to compounds 1, 2, 3, and 4 respectively. **(C,F,I,L)** Magnification within the whole spectrum of the extract of WF4 cultured on rice medium, showing the absence or presence of peaks corresponding to compounds 1, 2, 3, and 4 respectively.

Since the extract from endophyte WF4 had the most potent and the widest anti-fungal target spectrum, it was subjected to bio-assay guided purification using *F. graminearum* to isolate the active anti-fungal compound(s). The fermentation and extraction procedures were scaled up (see Materials and Methods) to permit purification and structural elucidation using two different solid medium (rice and millet). Flow charts that illustrate the purification procedures are included (Figures S1, S2). LC/MS analysis was conducted on the rice-derived extract (Figure 4A, Supplemental Table S3) and millet-derived extract (Figure 4B, Supplemental Table S4), and bioactive compounds eluted from the column were used as reference to track the retention time and relative abundance of these compounds relative to the total extract ingredients. The retention times (min) of each of the purified compounds were as follows: Compound 1: 14.63; Compound 2: 15.9; Compound 3: 15.98; Compound 4: 18.94 (Figure 4). Mass values (M+H^+^) of the purified antifungal compounds (Figures 5A,D,G,J) are shown, alongside the corresponding zoomed-in peaks from extracts of WF4 grown on either millet medium (Figures 5B,E,H,K) or rice medium (Figures 5C,F,I,L). The molecular weights of each of the purified compounds were as follows: Compound 1: 253; Compound 2: 197; Compound 3: 258; Compound 4: 272 (Figure 5). Compound 1 could be extracted from WF4 grown on both millet and rice (Figures 5B,C), whereas Compound 2 was observed on rice culture only (Figures 5E,F). Compounds 3 and 4 could be observed from millet culture only (Figures 5H,I,K,L). The purified compounds were then subjected to further spectroscopic structure elucidation. Detailed spectroscopic data for the purified compounds are included (1D-NMR, Supplemental Table S5; data for 2D-NMR are not shown). The IR, mass and 1D-NMR data were as follows:

### *Isolation of Compound 1* (isolated from rice culture and confirmed on millet cultures by LC-MS)

Colorless crystals with molecular formula, C_15_H_11_NO_3_ and molecular weight 253, precipitated from the ethyl acetate-methanol (95:5 v/v) fraction (2.5 g from 20 g total extract). Compound 1 was purified by crystallization. The crystallization process involved dissolving in minimum volume of chloroform, followed by drop wise addition of hexane until slight turbidity was achieved; the solution was left to re-crystallize at room temperature. The process was repeated three times for further purification, followed by final washing with hexane. The purified compound eluted as a single band on a glass TLC with an Rf value of 0.2 using a solvent mixture of ethyl acetate-hexane (v/v 80:20), and with an Rf value of 0.34 using 100% ethyl acetate as solvent. The IR, ^1^H NMR,^13^C NMR data were as following:

IR: 3861, 2395, 2172, 1637 cm^−1^.

^1^H NMR (600 MHz, DMSO): 12.2 (1H, s, OH), 9.5 (1H, s, OH), 9.1 (1H, s, NH), 7.34 (1H, dd, J = 8.0, 1.0 Hz), 7.32 (1H, ddd, J = 8.0, 8.0, 1.0 Hz), 7.29 (1H, dd, J = 8.0, 8.0 Hz), 7.09 (1H,dd, J = 8.0, 1.0 Hz), 7.07 (1H, ddd, J = 8.0, 8.0, 1.0 Hz), 6.82 (1H, m), 6.72 (1H, ddd, J = 8.5, 1.5, 1.0 Hz), 6.71 (1H, dd, J = 2.5, 1.5 Hz).

^13^C NMR (150 MHz, DMSO): 158.3 (C), 157.2 (C), 142.2 (C), 134.9 (C), 133.1 (C), 129.3 (CH), 126.4(CH), 124.4 (CH), 124.06 (C), 122.1 (CH), 120.9 (C), 120.3 (CH), 116.6 (CH), 115.2 (CH), 114.6 (CH).

**Figure 6:**
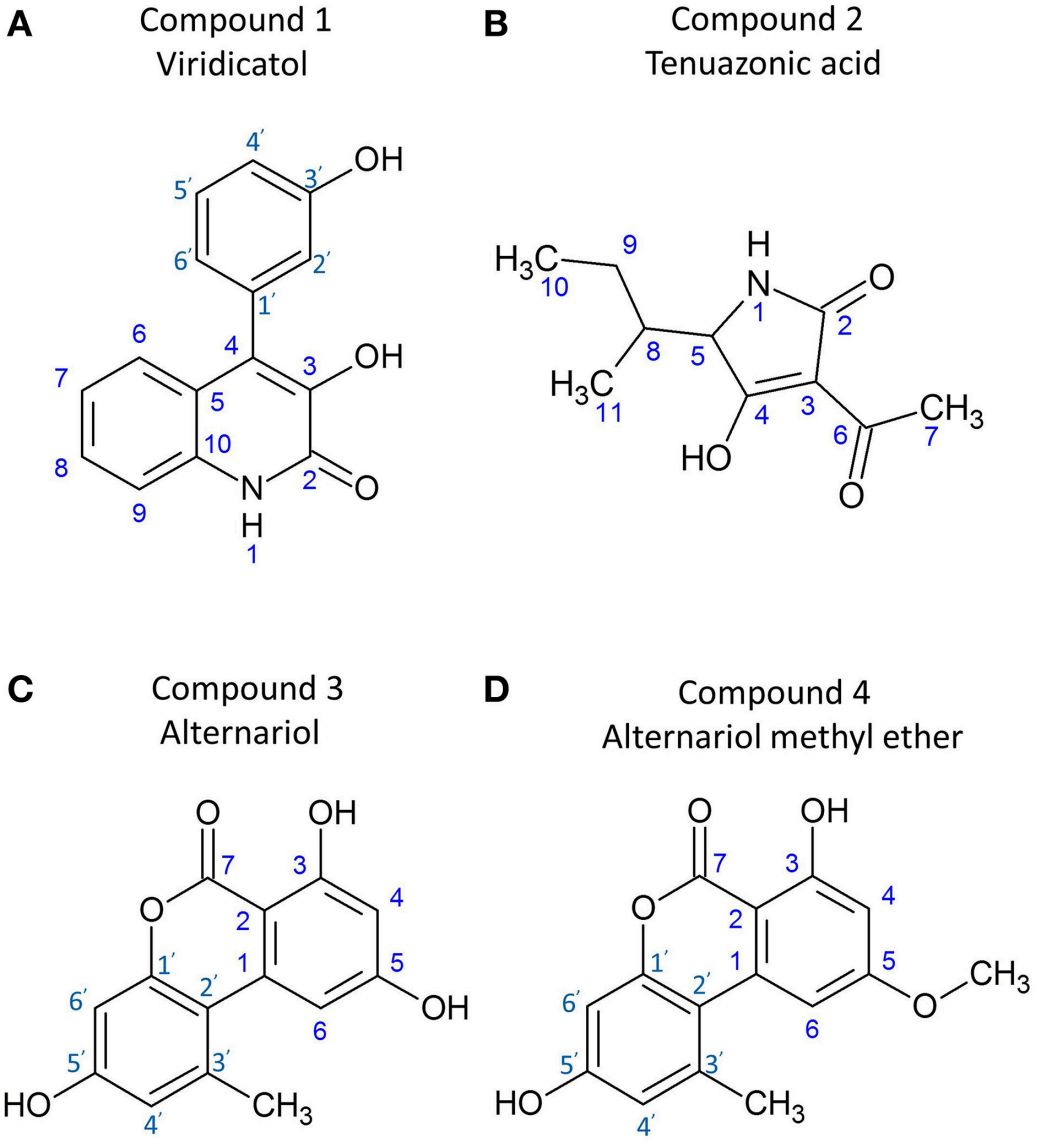
**Structures of the purified anti-***Fusarium*** compounds from endophyte WF4**. Shown are the structures for: **(A)** viridicatol alkaloid, **(B)** tenuazonic acid, **(C)** alternariol, and **(D)** alternariol monomethyl ether.

Comparing these results with reference data (Wei et al., 2011), the compound was confirmed as viridicatol alkaloid (3-hydroxy-4-(3-hydroxyphenyl)-2-quinolone monohydrate), in the 4-arylquinolin-2(1H)-ones class (Figure 6A).

### *Isolation of Compound 2* (isolated from rice culture)

A yellowish brown amorphous solid with molecular formula, C10H15O3N and molecular weight, 197 g/mol eluted at 52 min, yielding 15 mg from 250 mg. The IR, ^1^H NMR,^13^C NMR data were as follows:

IR: 3864, 3331, 2924, 2361, 1617 cm^−1^.

^1^H NMR (600 MHz, d4 methanol): 0.95 (t, J = 7.2 Hz, 3H, methyl), 1.01 (d, J = 6.9 Hz, 3H methyl), 1.34 (m, CH2), 1.89 (m, CH), 2.4 (s, 3H, methyl keto), 3.7 (d, CO-CH-N)

^13^C NMR (150 MHz, d4 methanol): 198.8 (C), 195.01 (C), 178.9 (C), 105.6 (CH), 67.20 (CH), 38.3 (CH), 27.06 (CH2), 24.26 (CH3), 16.65 (CH3), 12.42 (CH3)

Comparing these results with reference data (Mikula et al., 2013) the compound was confirmed as tenuazonic acid (3-acetyl-5-sec-butyl tetramic acid) (Figure 6B).

### *Isolation of Compound 3* (isolated from millet culture)

A white amorphous powder with molecular formula, C14H10O5 and molecular weight, 258 g/mol eluted at 22 min, yielding 8 mg from 250 mg. The IR, ^1^H NMR,^13^C NMR data were as follows:

IR: 3500, 2360, 1664, 1164 cm^−1^.

^1^H NMR (600 MHz, d4 methanol): 2.8 (3H, s, CH3), 7.3 (d, J = 1.8), 6.7 (d, J = 2.5), 6.6 (d, J = 2.5 Hz) and 6.4 (d, J=1.8).

^13^C NMR (150 MHz, d6 DMSO): 25.8 (CH3), 99 (C), 100.6(CH), 101.9 (CH), 104.3 (C), 109(C), 118.56 (CH), 139.8(CH), 140 (C), 154.4 (C), 159.8(C), 166.2(C), 166.8 (C), 167 (C).

Comparing these results with reference data (Tan et al., 2008; Ashour et al., 2011), the compound was confirmed as alternariol (Figure 6C).

### *Isolation of Compound 4* (isolated from millet culture)

White crystals with molecular formula, C15H12O5 and molecular weight, 272 g/mol, were eluted at 42 min, yielding 10 mg from 250 mg. The IR, ^1^H NMR,^13^C NMR data were as follows:

IR: 3320, 2925, 2854, 2360, 1666 cm^−1^.

^1^H NMR (600 MHz, d4 methanol): 2.8 (3H, s, CH3), 3.9 (3H, s, OCH3), 7.3 (d, J = 1.8), 6.7 (d, J = 2.5), 6.6 (d, J = 2.5 Hz) and 6.59 (d, J = 1.8).

^13^C NMR (150 MHz, d6 DMSO): 25.5 (CH3), 56.3 (OCH3), 98.9 (C), 99.6(CH), 102 (CH), 103.8 (C), 109(C), 118.1 (CH), 138.2(CH), 138.8 (C), 153 (C), 166.6(C), 164.6(C), 165.1 (C), 166.6 (C).

Comparing these results with reference data (Ashour et al., 2011), the compound was confirmed as alternariol-5-O-methyl ether or djalonensone (3,7-Dihydroxy-9-methoxy-1-methyl-6H-dibenzo[b,d]pyran-6-one) (Figure 6D).

### Effects of pure endophyte-derived compounds on *F. graminearum in vitro*

**Figure 7:**
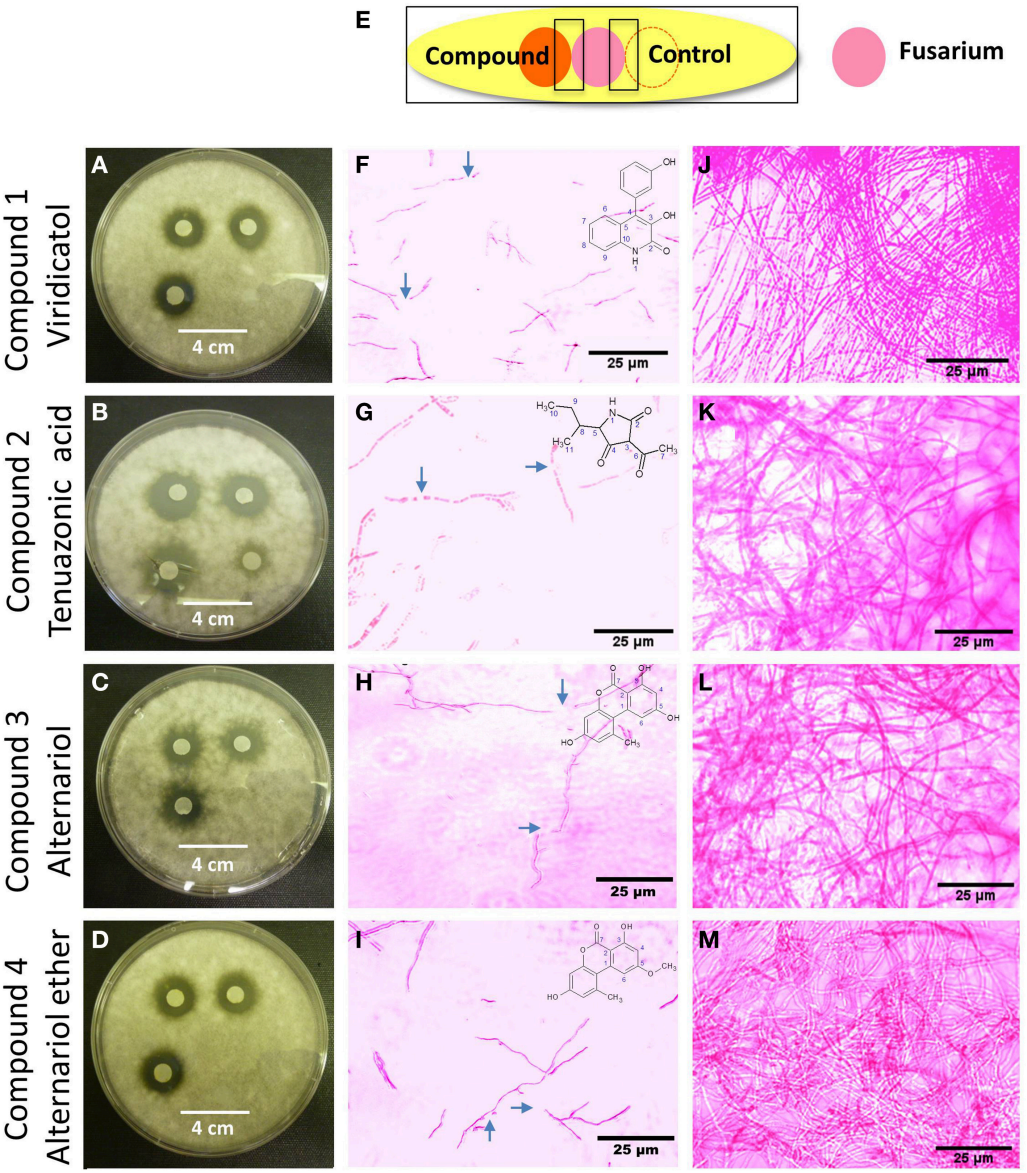
**The effects of the purified compounds on ***F. graminearum in vitro*** using the agar disc diffusion assay and neutral red staining**. **(A–D)** Representative pictures of the agar disc diffusion assay of the purified anti-fungal compounds, **(A)** viridicatol, **(B)** tenuazonic acid, **(C)** alternariol, and **(D)** alternariol monomethyl ether (*n* = 3). The inhibition of *F. graminearum* is shown by the clear halo around each disc soaked with the purified compound. **(E)** Cartoon of the experimental methodology to examine microscopic *in vitro* interactions between *F. graminearum* (pink) and each compound (orange) or the buffer control (respective compound solvent). The microscope slides were pre-coated with PDA and incubated for 24 h. *F. graminearum* hyphae were then stained with the vitality stain, neutral red. Shown are representative microscope slide pictures (*n* = 3) of the interactions of *F. graminearum* with: **(F)** viridicatol compared to **(J)** the buffer control; **(G)** tenuazonic acid compared to **(K)** the buffer control; **(H)** alternariol compared to **(L)** the buffer control; **(I)** alternariol monomethyl ether compared to **(M)** the buffer control. The blue arrows point to areas of apparent breakage of *F. graminearum* hyphae.

The putative anti-fungal compounds, viridicatol, tenuazonic acid, alternariol and alternariol-mono methyl ether were verified as having anti-*F. graminearum* activity by using the agar disc diffusion method (diameter of fungal inhibition zone): application of the compounds 1–4 (20 μl of 5 mg/ml) caused inhibition zones of 1.8, 2, 1.5, 1.5 mm (respectively) compared to the solvent buffer (0–0.5 mm) (Figures 7A–D), respectively.

**Figure 8:**
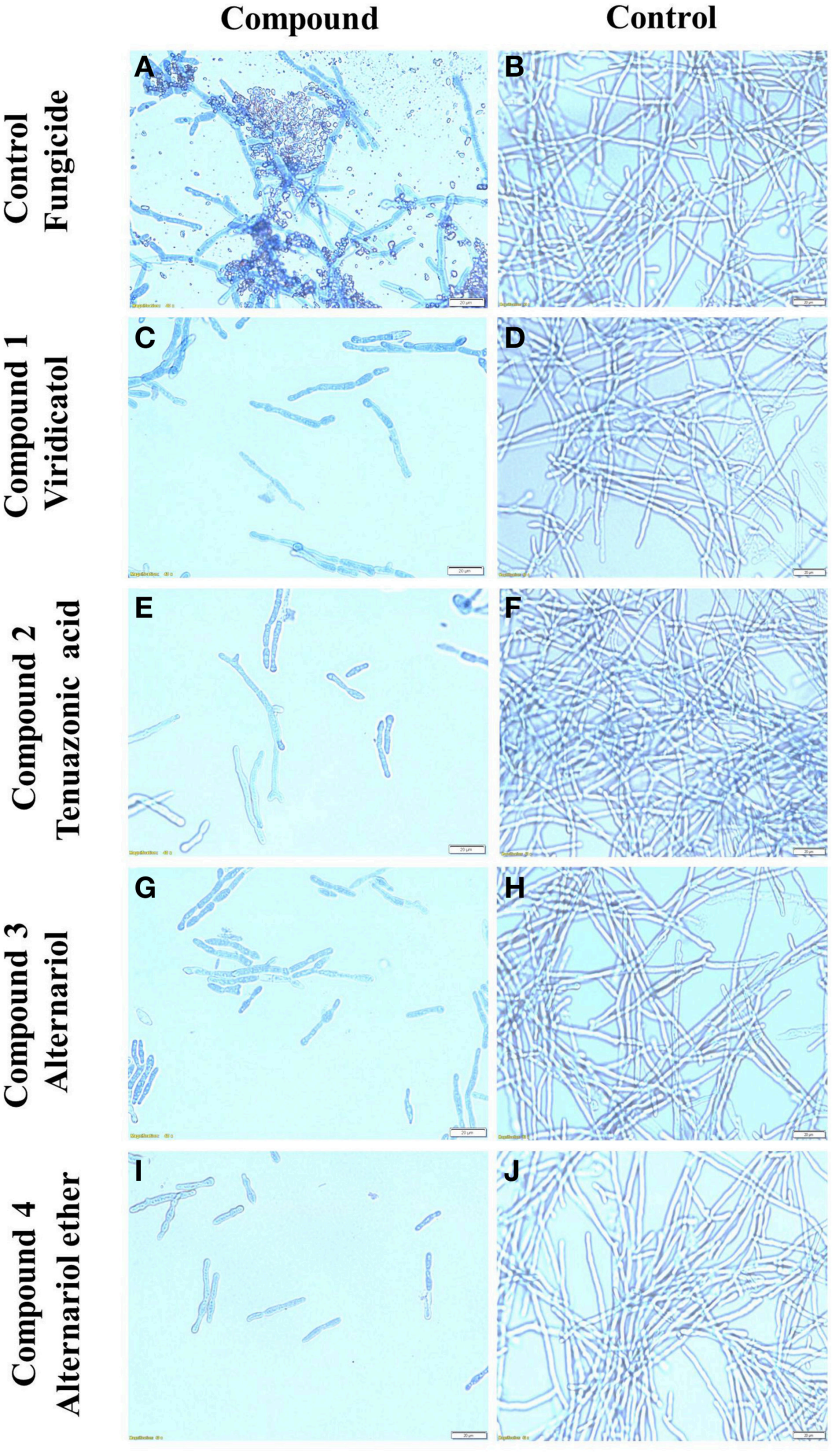
**The effects of the purified compounds on ***F. graminearum in vitro*** using Evans blue staining**. Shown are representative microscope slide pictures (*n* = 3) of the interactions of *F. graminearum* with: **(A)** the fungicide PROLINE compared to **(B)** the buffer control; **(C)** viridicatol compared to **(D)** the buffer control; **(E)** tenuazonic acid compared to **(F)** the buffer control; **(G)** alternariol compared to **(H)** the buffer control; **(I)** alternariol monomethyl ether compared to **(J)** the buffer control. In **(A)**, the aggregated particles between the hyphae are precipitates of PROLINE.

The purified compounds (20 μl of 5 mg/ml) were co-incubated for 24 h with *F. graminearum* then stained with neutral red or Evans blue (stains dead cells blue), to examine the *in vitro* microscopic interactions between the compounds and the pathogen (Figure 7E). All the compounds caused apparent breakage of the *F. graminearum* hyphae (Figures 7F–I) compared to the buffer controls (Figures 7J–M), respectively. Similar to the Proline fungicide control (Figure 8A), the fungal hyphae adjacent to each compound stained blue with Evans blue (Figures 8C,E,G,I) compared to buffer controls (Figures 8B,D,F,H,J), suggesting that hyphae in contact with each endophytic compound died.

## Discussion

We hypothesized that finger millet may host endophytes with anti-fungal activity, including against the pathogenic species of *Fusarium*, because this crop is known to be resistant to many pathogens (Munimbazi and Bullerman, 1996), including *Fusarium* ssp. pathogens of Africa where the crop was domesticated (Adipala, 1992; Amata et al., 2010; Saleh et al., 2012) and South Asia (Penugonda et al., 2010; Ramana et al., 2012). To the best of our knowledge, this is the first report of endophyte(s) from finger millet. The putative endophytes were isolated from plants from first generation seeds from India, and then grown on Turface clay rock using hydroponics rather than on soil, suggesting they did not derive from Canadian soil. This growth system might explain why mycorrhizal fungi were also not isolated. Here, extracts from three of the five fungal endophytes (WF4, WF6, and WF7) isolated from finger millet roots inhibited growth of *F. graminearum in vitro*, along with six other fungal pathogens (*Alternaria alternata, Aspergillus flavus, Fusarium lateritium, Fusarium sporotrichioides, Fusarium avenaceum*, and *Trichoderma longibrachiatum*), including three other pathogenic *Fusarium* species (Table 1). The extract from endophyte WF4 (predicted to be a *Phoma* sp.) had the widest anti-fungal target spectrum (7/26 fungal pathogens tested, Table 1) and was the most potent of the extracts against *F. graminearum* (Figure 1I). Furthermore, the extract from endophyte WF4 inhibited the growth of four tested *Fusarium* pathogens (Table 1). For these reasons, we decided to focus the current study on this endophyte. Inoculation of finger millet seedlings with WF4 resulted in no disease symptoms (Figure 2); furthermore, Calcofluor staining and confocal microscopy imaging confirmed the ability of WF4 to efficiently colonize root tissues (Figure 3). Combined with the fact that this fungus was initially isolated from surface sterilized plants, these results confirmed the endophytic behavior of WF4 in finger millet. Bio-guided isolation of the active antifungal compounds from the extract of WF4 were conducted using two fermentation media, rice and millet. Inactive fractions, including those that might have compounds active against other fungi, were not subjected to further study. The structural elucidation of the active anti-*Fusarium* compounds from WF4 revealed two compounds when the fungus was cultured on rice (viridicatol and tenuazonic acid), and three compounds when cultured on millet (viridicatol, alteraniol and alteraniol methyl ether) (Figures 4–6) and (Figures S1, S2). As different media are enriched in different metabolic precursors, these results were not surprising, as previously shown (Aly et al., 2008). Mechanistically, all the purified compounds caused dramatic breakage of *F. graminearum* hyphae (Figures 7, 8).

### Anti-fungal compounds previously isolated from *Phoma* species

The focus of this study was endophytic fungal strain WF4, predicted to be a *Phoma* sp. *Phoma* species have previously been shown to be endophytes of other plants. For example, a *Phoma* species was isolated as an endophyte from the Chinese medicinal plant *Arisaema erubescens* (Wang et al., 2012), from the Chinese medicinal plant *Taraxacum mongolicum* Hand.-Mazz (Zhang et al., 2013b), a Tibetan medicinal plant *Phlomis younghusbandii* Mukerjee (Zhang et al., 2013a) and from *Acer ginnala* Maxim, a tree from northeastern China (Qi et al., 2012).

Consistent with our study, anti-fungal compounds have previously been isolated from *Phoma* species. For example, the compound phomalone, purified from *Phoma etheridgei*, which was isolated from black galls of Canadian aspen trees, showed antifungal activity against *Phellinus tremulae* (an aspen decay fungus) (Ayer and Jimenez, 1994). In another study, an α-tetralone derivative, (3*S*)-3,6,7-trihydroxy-α-tetralone, was purified from a *Phoma* endophyte, isolated from the Chinese medicinal plant *Arisaema erubescens*, and shown to have antifungal activities against *Fusarium oxysporum* and *Rhizoctonia solani* (Wang et al., 2012). The polyketide, 5-hydroxyramulosin, isolated from an endophytic fungus morphologically similar to *Phoma*, inhabiting the tree *Cinnamomum mollissimum* from Malaysia, was shown to have antifungal activity against *Aspergillus niger* (Santiago et al., 2012). The compounds, epoxydines A and B, epoxydon, (4R,5R,6S)-6-acetoxy-4,5-dihydroxy-2-(hydroxymethyl)cyclohex - 2-en-1-one and 2-chloro-6-(hydroxymethyl)benzene-1,4-diol, were purified from a *Phoma* sp. isolated from *Salsola oppositifolia* (Amaranth family) following fermentation, and shown to have antifungal activities against the fungus *Microbotryum violaceum* (Qin et al., 2010). Finally, the polyketide xanthepinone, a metabolite with antifungal activity against the fungal pathogens, *Pyricularia oryzae, Botrytis cinerea* and *Phytophthora infestans*, was purified from a fungus classified as a *Phoma* sp. which was collected from French Alpine soil (Liermann et al., 2009). Our study further suggests that it may be useful to further explore diverse *Phoma* species for new anti-fungal compounds.

### Viridicatol

In this study, we identified the alkaloid viridicatol as one of active anti-fungal compounds from WF4 endophyte extracts fermented on both rice and millet media (Figures 4A, 5A–C). The ring system of viridicatol is synthesized from phenylalanine and anthranilic acid (precursor to auxin and tryptophan) (Luckner and Mothes, 1962), from benzodiazepine derivatives (Luckner et al., 1969). Viridicatol was shown to be cytotoxic toward various tumor cell lines (Wei et al., 2011). The related compound viridicatin (di-hydroxylated version of viridicatol) was first described in 1953, isolated from *Penicillium viridicatum* Westling (Cunningham and Freeman, 1953) and subsequently shown to be produced by different *Penicillium* spp. (Frisvad et al., 2004). Viridicatol has also been reported outside of the *Penicillium* clade, as it has been purified from *Aspergillus versicolor* from beach sand in Australia (Fremlin et al., 2009) and from a marine isolate of *A. versicolor* from Sakhalin Bay, Russia (Yurchenko et al., 2010). Emerging from the literature is that viridicatol is primarily produced by fungi that survive harsh environmental conditions such as pathogen exposure (Cunningham and Freeman, 1953; Sutton, 1982; Payne and Widstrom, 1992) and in extreme environments such as the Mir space station (Kozlovskii et al., 2002) and mangroves (Wei et al., 2011). These observations are consistent with this study, in which viridicatol was isolated from an endophyte of finger millet, a crop that is adapted to extreme stress, including heat, drought and low soil fertility (Uma et al., 1995). To the best of our knowledge, this is the first report of viridicatol from a *Phoma* species, and the first report of viridicatol having anti-fungal activity. Understanding the full ecological role of viridicatol may come from knocking out or over-expressing the biosynthetic genes that encode this compound.

### Tenuazonic acid

In this study, we identified tenuazonic acid (3-Acetyl-1,5-dihydro-4-hydroxy-5-(1- methylpropyl)-2H-pyrrol-2-one) a tetramic acid analog, as one of the active anti-fungal compounds from the extract of WF4 grown on rice medium (Figures 4B, 5D,F). However, the compound was not detected when the fungus was fermented on millet medium (Figure 5E), suggestive of an important underlying plant-endophyte interaction (biochemical precursor or inducer/repressor). Tenuazonic acid is a potent mycotoxin belonging to the 3-acylpyrrolidine-2,4-diones family of tetramic acid analogs (Weidenbörner, 2001). It is biosynthesized from L-isoleucine in *Alternaria tenuis* (Gatenbeck and Sierankiewicz, 1973). In previous studies, tenuazonic acid showed antibacterial, antiviral and antitumor activities (Miller et al., 1963; Gitterman, 1965). It also inhibited the growth of human adenocarcenoma (Kaczka et al., 1963). Mechanistically, tenuazonic acid suppresses the release of newly formed peptides from the ribosome, resulting in inhibition of protein synthesis (Shigeura and Gordon, 1963). However, the practical application of the compound is restricted due to its extreme toxicity. The compound was reported to be produced by various fungi including *Alternaria alternata, A. tenuissima* (Davis et al., 1977), *Pyricularia oryzae* (Iwasaki et al., 1972), *Aspergillus* sp. (Miller et al., 1963), and *Phoma sorghina* (Steyn and Rabie, 1976). To the best of our knowledge, this is the first report that tenuazonic acid has anti-*Fusarium* activity.

### Alternariol and alternariol monomethyl ether

In this study, we identified alternariol and alternariol monomethyl ether as anti-fungal polyketide compounds produced by endophyte WF4 when cultured on millet medium (Figures 4C,D, 5G,H,J,K). However, these compounds were not detected when the fungus was fermented on rice medium (Figures 5I,L), again suggestive of an underlying plant-endophyte interaction. These benzopyranone derivatives are biosynthesized from acetyl-CoA and malonyl-CoA via the polyketide pathway (Tanahashi et al., 2003). Alternariol derivatives are typical examples of the dibenzo-α-pyrones family, members of which are key intermediates in the biosynthesis of pharmacologically active compounds such as cannabinoids (Teske and Deiters, 2008) and progesterone (Edwards et al., 1998). They were reported to be toxic to chickens (Ostry, 2008) and brine shrimp (Zajkowski et al., 1991). Alternariol exhibits remarkable cytotoxicity against L5178Y mouse lymphoma cells (Aly et al., 2008). Further studies showed that alternariol and its monomethyl ether could inhibit topoisomerase I and II by interchelating DNA in human carcinoma cell lines (Fehr et al., 2009). Alternariol monomethyl ether exhibited anti-nematodal activity against *Caenorhabditis elegans* and inhibited spore germination of the rice blast fungus, *Magnaporthe oryzae* (Meng et al., 2012). Alternariol monomethyl ether (djalonensone) was previously isolated from the roots of the west African medicinal plant *Anthocleista djalonensis* and claimed to be a taxonomic marker of this plant species (Onocha et al., 1995). However, some debates have been raised as to whether this metabolite might actually be produced by an endophytic fungus inhabiting it (such as *Acremonium* sp.) not the plant itself (Hussain et al., 2007). The dibenzo-α-pyrones family is mainly produced by *Alternaria* species in addition to some other fungi such as *Hyalodendriella* sp., *Cephalosporium acremonium, Microsphaeropsis olivacea, Penicillium verruculosum, Botrytis allii* TC 1674, and *Phoma* sp. (Mao et al., 2014). To the best of our knowledge, this is the first report of alternariol and alternariol monomethyl ether from a *Phoma* sp., and the first report of them having anti-*Fusarium* activity.

### Study limitations and future experiments

There were some limitations of this study. First, this study confirmed the endophytic behavior of only one of the fungal strains isolated (WF4); more detailed studies of the other strains will be reported in future papers. Second, only 18S rDNA sequences were used for taxonomic identification of the putative fungal endophytes; as multiple fungal species can share the same 18S sequence (Reiss et al., 1997), the identification of these endophytes will require further validation from additional DNA sequencing. Third, the results reported in this study were based on *in vitro* assays only, and further studies will be required to test their relevance to the natural plant habitat. In particular, a separate study is required to determine whether the dosages used here *in vitro* (5 mg/ml) are relevant *in planta* as they are relatively high compared to the average dosage used for commercial fungicides (Reis et al., 2015). According to previous research, *in vitro* dosage data has only limited value for predicting field dosages, which varies by fungicide (Reis et al., 2015). This lack of *in vitro* vs. field dosage correlation might be due to variation between fungicides in their uptake, stability under field conditions or degree of degradation by the plant. Field application requires *in planta* testing followed by further studies to increase the potency/stability of a fungicide, perhaps by chemical/biological derivatization (Kirschning and Hahn, 2012). Similar to the challenge faced by natural products chemists, it is currently not possible to purify these endophytic chemicals in sufficient amounts for *in planta* testing, especially on large crops. However, these natural products could be used for future synthesis and semi-synthesis as lead compounds (Kobayashi and Harayama, 2009). As for testing of the putative endophytes themselves *in planta*, finger millet is not susceptible to *F. graminearum*, so future experiments would need to employ a heterologous host, but the endophytes may not colonize a non-host plant.

We suggest some additional future experiments of interest. First, given that we were able to isolate several putative endophytes from only a single finger millet genotype, it will be valuable to bioprospect diverse finger millet genotypes from Africa and South Asia including ancient and ancestral varieties (Goron and Raizada, 2015). One of the available resources for conducting this type of research is the International Crops Research Institute for the Semi-Arid Tropics (ICRISAT) which has a large collection of finger millet landraces from around the world (http://www.icrisat.org/) (Goron and Raizada, 2015). In a previous study, we demonstrated that different genotypes of maize possess a wide diversity of endophytes with potentially useful traits (Johnston-Monje and Raizada, 2011b). It may be useful to test this potential diversity of finger millet endophytes for their ability to combat not only *Fusarium* species, including African isolates of *Fusarium verticilliodes*, but also *Pyricularia oryzae*, the fungal agent responsible for blast disease, to which finger millet is highly susceptible (Yaegashi and Asaga, 1981). However, as finger millet is susceptible to *P. oryzae*, it seems less likely that the putative endophytes isolated here would have anti-*P. oryzae* activity. Second, prior to using these endophytes or their compounds as commercial biocontrol agents, more detailed experiments are needed to examine the toxicities and safety of the endophyte derived extracts, including at different concentrations, their effects on mammalian models and their persistence in ecosystems. Preliminary experiments using the extract from endophyte WF4 fermented on rice medium showed that it causes variable degrees of toxicity against leaf discs, fruit flies and seeds (Figure S3 and Supplemental Methods). Third, as *F. graminearum* has the potential to cause root rot in the seedlings of diverse cereals (Chongo et al., 2001), it is interesting to speculate whether the seedling root endophytes isolated from this study suppress *Fusarium* root disease in finger millet; this speculative hypothesis will require future testing but might suggest the biological relevance of these endophytes, given that *Fusarium* diseases in other cereals are most common in shoots and inflorescences.

## Conclusions

To the best of our knowledge, this is the first report of endophyte(s) from finger millet. Here, putative fungal endophytes were isolated from root tissues of Indian finger millet. *In vitro*, the endophytic extracts showed activity against diverse fungi, including multiple pathogenic species. Using a bio-guided assay, we purified four anti-fungal compounds from two different cultures of the most potent anti-fungal strain (strain WF4, predicted to be a *Phoma* sp.), which was confirmed to behave as an endophyte. Of these compounds, viridicatol, alternariol and alternariol monomethyl ether have not been reported to be produced by *Phoma* previously. The mode of action of all of these compounds involves apparent breakage of *Fusarium* hyphae. This study suggests that exploration of endophytes in ancient, subsistence crops may result in the identification of organisms or chemicals with potential to control the diseases of modern crops. These crops have been grown continuously without pesticides including fungicides. We hope the results presented here will encourage other researchers to explore the diversity of finger millet and other ancient crops which have been largely ignored by many Western scientists. Practical application of the endophytic natural products identified from this study will require *in planta* experiments at the field level.

### Conflict of interest statement

The authors declare that the research was conducted in the absence of any commercial or financial relationships that could be construed as a potential conflict of interest.

## Supplementary material

Figure S1 **Flow chart illustrating the bio-guided purification of the active anti-Fusarium compounds from the extract of endophyte WF4 grown on rice culture**.

Figure S2 **Flow chart illustrating the bio-guided purification of the active anti-Fusarium compounds from the extract of endophyte WF4 grown on millet culture**.

Figure S3 **Preliminary toxicity assays of the extract from endophyte WF4. (A,B)** Leaf punch toxicity assay: **(A)** The methodology used to test the extract for its ability to cause lesions on maize leaf punches over a 6 day period (*n* = 4 per dilution). **(B)** Quantification of the leaf lesions caused by different dilutions of the extract, following 2, 4, and 6 days of co-incubation (*n* = 4). **(C,D)** Fruit fly toxicity assay: **(C)** Picture showing the different developmental stages of the fly: larva, pupa and adult. **(D)** Quantification of the effect of the extract or the buffer control on the onset, duration and percentage (relative to starting eggs) of each developmental stage. **(E–H)** Seed germination toxicity assay: **(E)** Representative pictures of corn seeds on filter paper exposed to the endophyte extract compared to exposure to the buffer. **(F)** Quantification of the effect of different extract aqueous dilutions (100, 80, 50% dilutions) or the buffer control, on the germination of corn seeds. **(G)** Representative pictures of wheat seeds on filter paper exposed to the endophyte extract compared to exposure to the buffer control. **(H)** Quantification of the effect of different extract aqueous dilutions (100, 80, 50% dilutions) or the buffer control on the germination of wheat seeds. For all of the toxicity assays, an asterisk indicates that the mean is significantly different than the control (*p* = 0.05, Mann-Whitney, *n* = 6 unless otherwise indicated). The error bars represent the standard error of the mean (SEM). See Supplemental Methods for more information.

Supplemental Table S1 **18S rDNA sequences and BLAST analysis of fungal endophytes isolated from finger millet in this study (corresponding to Table 1)**.

Supplemental Table S2 **Taxonomic identification of the isolated endophytic fungi from finger millet**.

Supplemental Table S3 **List of retention times and expected masses of compounds from extract of WF4 fungus fermented on rice**.

Supplemental Table S4 **List of retention times and expected masses of compounds from extract of WF4 fungus fermented on millet**. The antifungal compounds are highlighted in yellow.

Supplemental Table S5 **NMR data for atomic assignments of compounds isolated from WF4 fungal extracts**. The table includes compounds from WF4 fungi fermented on both rice and finger millet.
