# Supplementary figures and images for: An endophytic fungus isolated from finger millet (Eleusine coracana) produces anti-fungal natural products
Source: Front Microbiol. 2015 Oct 21;6:1157. doi: 10.3389/fmicb.2015.01157 (PMC4612689; doi:10.3389/fmicb.2015.01157)

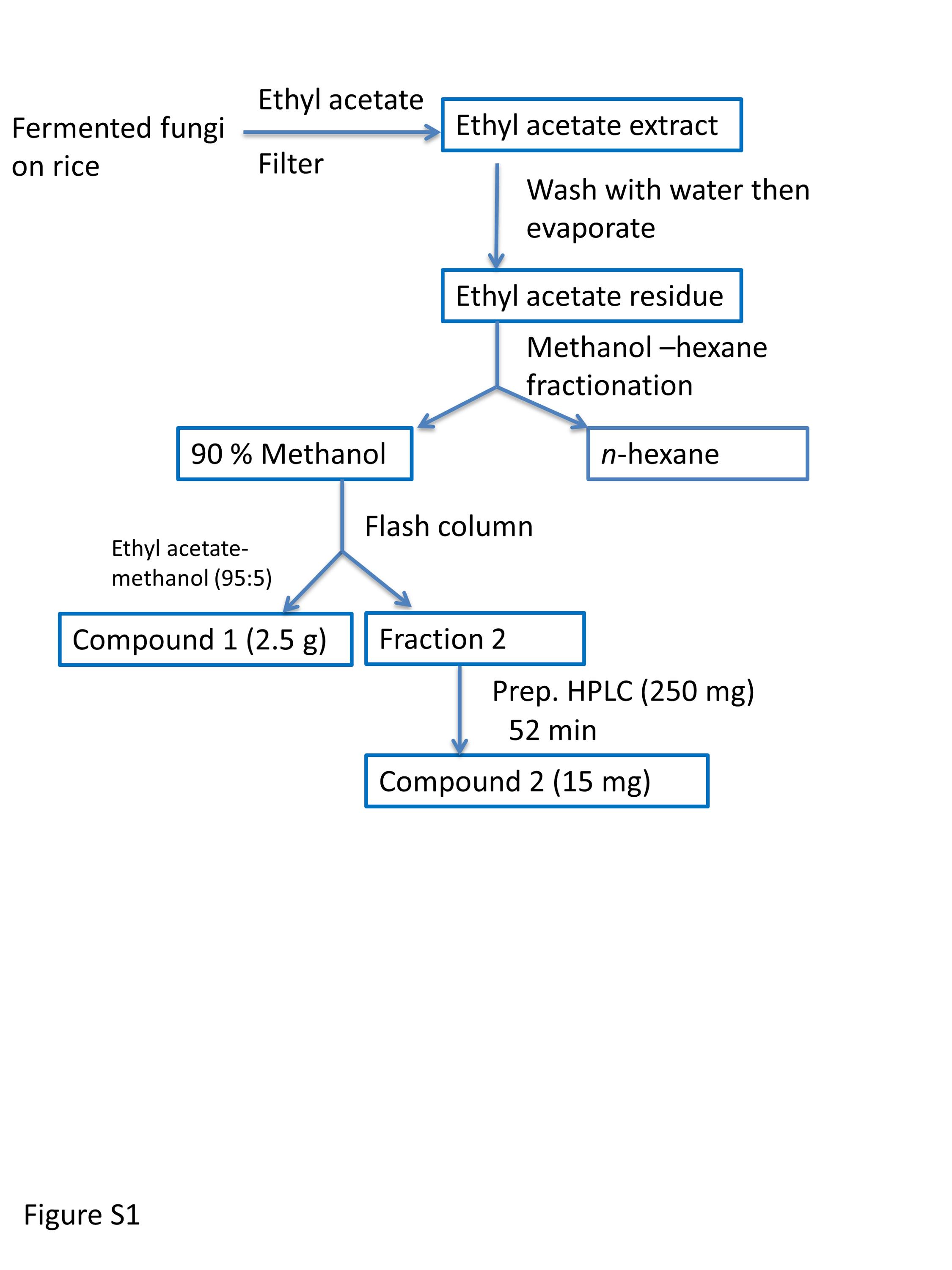

Supplement: Figure S1 — Flow chart illustrating the bio-guided purification of the active anti-Fusarium compounds from the extract of endophyte WF4 grown on rice culture. [file Image1.TIF]

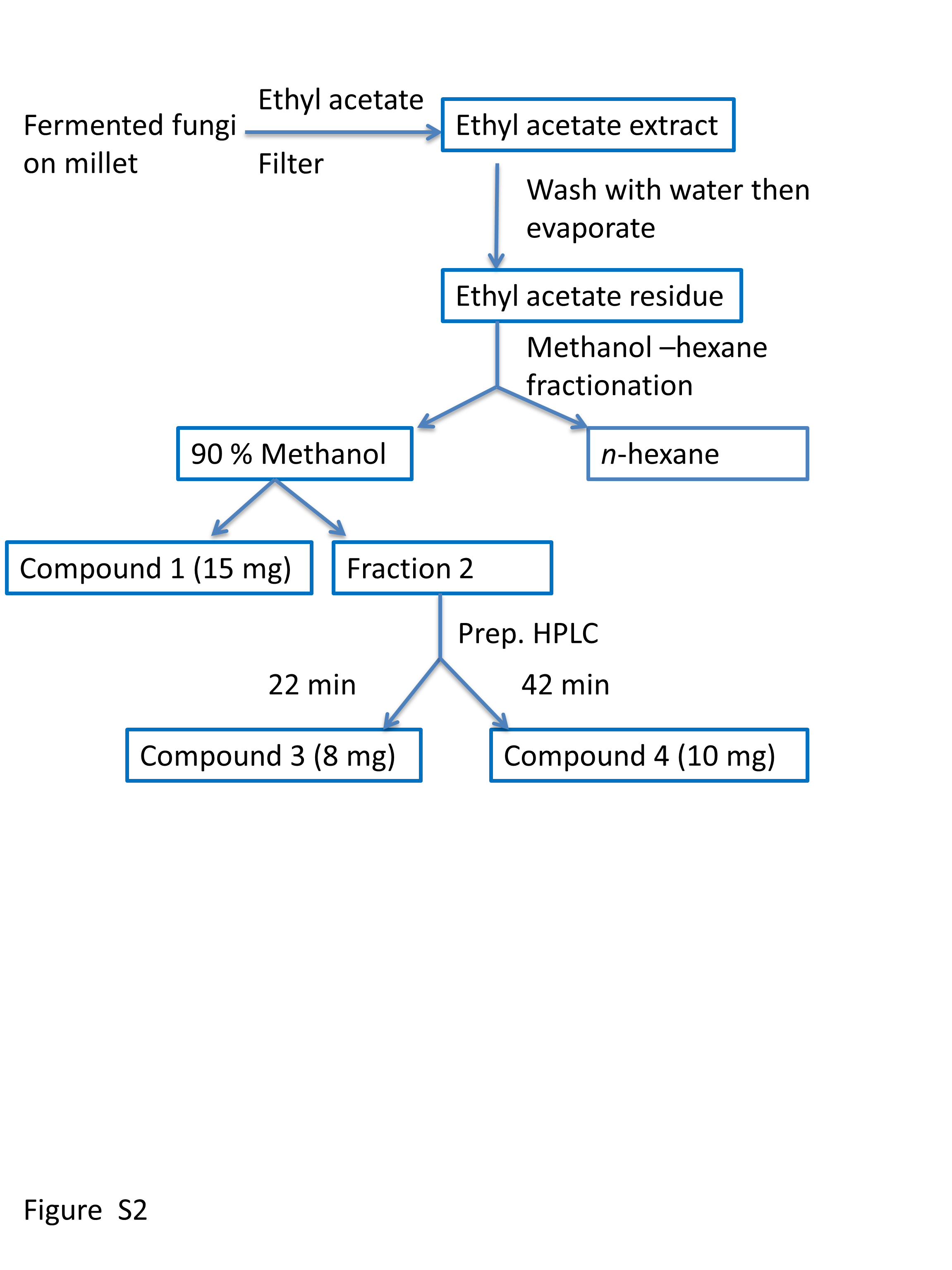

Supplement: Figure S2 — Flow chart illustrating the bio-guided purification of the active anti-Fusarium compounds from the extract of endophyte WF4 grown on millet culture. [file Image2.TIF]

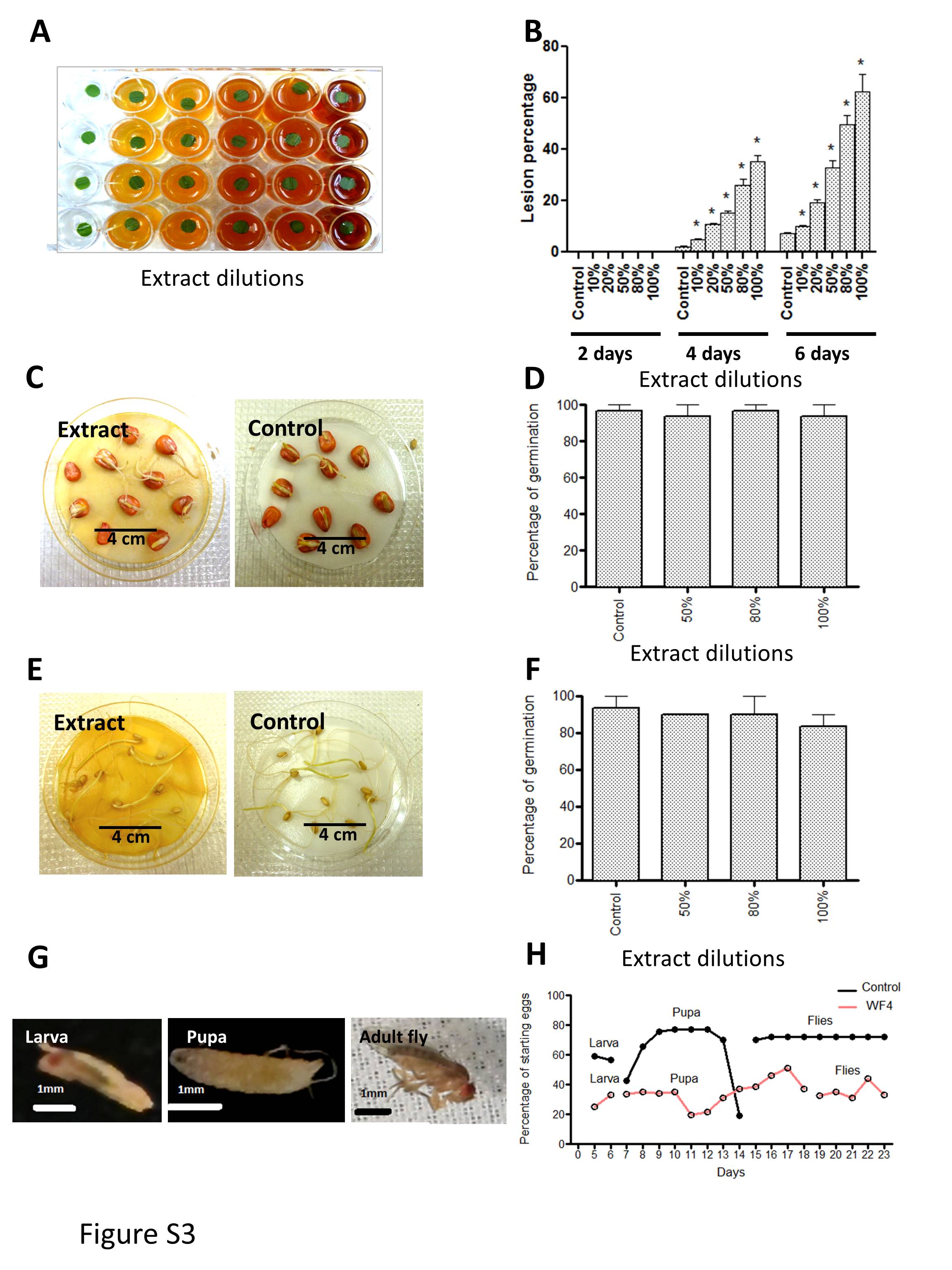

Supplement: Figure S3 — Preliminary toxicity assays of the extract from endophyte WF4. (A,B) Leaf punch toxicity assay: (A) The methodology used to test the extract for its ability to cause lesions on maize leaf punches over a 6 day period (n = 4 per dilution). (B) Quantification of the leaf lesions caused by different dilutions of the extract, following 2, 4, and 6 days of co-incubation (n = 4). (C,D) Fruit fly toxicity assay: (C) Picture showing the different developmental stages of the fly: larva, pupa and adult. (D) Quantification of the effect of the extract or the buffer control on the onset, duration and percentage (relative to starting eggs) of each developmental stage. (E–H) Seed germination toxicity assay: (E) Representative pictures of corn seeds on filter paper exposed to the endophyte extract compared to exposure to the buffer. (F) Quantification of the effect of different extract aqueous dilutions (100, 80, 50% dilutions) or the buffer control, on the germination of corn seeds. (G) Representative pictures of wheat seeds on filter paper exposed to the endophyte extract compared to exposure to the buffer control. (H) Quantification of the effect of different extract aqueous dilutions (100, 80, 50% dilutions) or the buffer control on the germination of wheat seeds. For all of the toxicity assays, an asterisk indicates that the mean is significantly different than the control (p = 0.05, Mann-Whitney, n = 6 unless otherwise indicated). The error bars represent the standard error of the mean (SEM). See Supplemental Methods for more information. [file Image3.JPEG]
