# Supplementary material for: An endophytic fungus isolated from finger millet (Eleusine coracana) produces anti-fungal natural products
Source: Front Microbiol. 2015 Oct 21;6:1157. doi: 10.3389/fmicb.2015.01157 (PMC4612689; doi:10.3389/fmicb.2015.01157)
Supplement: Supplemental Table S2 — Taxonomic identification of the isolated endophytic fungi from finger millet. [file Table2.DOCX]

**Supplemental Table S2. Taxonomic identification of the isolated endophytic fungi from finger millet.**

| **Endophyte Source** | **ID** | **Best BLAST Match** | **BLAST**  **E-value** | **Percent Maximum Identity** |
| --- | --- | --- | --- | --- |
| Root | WF1 | *Aspergillus niger* | 0.0 | 100% |
| Root | WF3 | *Penicillium griseofulvum* | 0.0 | 99% |
| Root | WF4 | *Phoma sp.* | 0.0 | 99% |
| Root | WF6 | [*Penicillium chrysogenum*](http://blast.ncbi.nlm.nih.gov/Blast.cgi#alnHdr_409029681) | 0.0 | 99% |
| Root | WF7 | *Penicillium expansum* | 0.0 | 99% |
