# Supplementary material for: An endophytic fungus isolated from finger millet (Eleusine coracana) produces anti-fungal natural products
Source: Front Microbiol. 2015 Oct 21;6:1157. doi: 10.3389/fmicb.2015.01157 (PMC4612689; doi:10.3389/fmicb.2015.01157)
Supplement: Supplemental Table S3 — List of retention times and expected masses of compounds from extract of WF4 fungus fermented on rice. [file Table3.DOCX]

| **RT** | **Mass** | **Height** |
| --- | --- | --- |
| 19.04 | 310.1762 | 11379 |
| 18.62 | 615.2709 | 21728 |
| 18.09 |  | 50974 |
| 17.4 | 237.0778 | 92309 |
| 17.35 | 496.137 | 13106 |
| 17.02 |  | 34135 |
| 15.9 | 197.1041 | 55204 |
| 15.9 |  | 20229 |
| 15.86 | 241.2755 | 23456 |
| 15.81 | 197.1042 | 10361 |
| 15.76 | 394.2085 | 170659 |
| 15.4 | 225.1713 | 14270 |
| 15.3 | 642.2621 | 18533 |
| 14.8 |  | 17886 |
| 14.67 | 506.145 | 123545 |
| 14.65 | 528.1266 | 65754 |
| 14.58 | 267.0881 | 72341 |
| 14.54 | 253.0726 | 1200810 |
| 14.39 | 296.1716 | 47065 |

**Supplemental Table S3.** List of retention times and expected masses of compounds from extract of WF4 fungus fermented on rice. The antifungal compounds are highlighted in yellow.
