# Supplementary material for: An endophytic fungus isolated from finger millet (Eleusine coracana) produces anti-fungal natural products
Source: Front Microbiol. 2015 Oct 21;6:1157. doi: 10.3389/fmicb.2015.01157 (PMC4612689; doi:10.3389/fmicb.2015.01157)
Supplement: Supplemental Table S4 — List of retention times and expected masses of compounds from extract of WF4 fungus fermented on millet. The antifungal compounds are highlighted in yellow. [file Table4.DOCX]

**Supplemental Table S4.** List of retention times and expected masses of compounds from extract of WF4 fungus fermented on millet. The antifungal compounds are highlighted in yellow.

| **RT** | **Mass** | **Height** |
| --- | --- | --- |
| 19.2 | 329.2929 | 1121689 |
| 19.05 | 714.4097 | 298849 |
| 19.02 | 328.2038 | 208142 |
| 18.94 | 272.0682 | 615910 |
| 18.43 | 311.2824 | 233954 |
| 18.38 | 742.3683 | 341327 |
| 18.36 | 360.2114 | 237420 |
| 18.06 | 340.3195 | 389414 |
| 17.96 | 709.5118 | 498328 |
| 17.15 | 315.2769 | 311725 |
| 17.12 | 288.0631 | 455058 |
| 16.52 | 348.2294 | 241596 |
| 15.98 | 258.0527 | 198187 |
| 15.95 | 320.1623 | 266682 |
| 15.92 | 416.2422 | 206654 |
| 15.81 | 358.2829 | 307812 |
| 15.62 | 364.2247 | 514146 |
| 15.61 | 414.2264 | 1282090 |
| 15.18 | 288.0634 | 220648 |
| 15.09 | 272.0681 | 324955 |
| 15.07 | 290.0789 | 395867 |
| 14.63 | 508.1538 | 310107 |
| 14.63 | 253.0736 | 795146 |
| 13.39 | 364.2239 | 201331 |
| 13.24 | 278.0785 | 196535 |
| 13.24 | 260.068 | 624325 |
| 12.64 | 234.0888 | 332494 |
| 12.44 | 402.0942 | 184080 |
| 12.22 | 234.0523 | 226496 |
| 12.09 | 252.0629 | 181700 |
