# Supplementary material for: An endophytic fungus isolated from finger millet (Eleusine coracana) produces anti-fungal natural products
Source: Front Microbiol. 2015 Oct 21;6:1157. doi: 10.3389/fmicb.2015.01157 (PMC4612689; doi:10.3389/fmicb.2015.01157)
Supplement: Supplemental Table S5 — NMR data for atomic assignments of compounds isolated from WF4 fungal extracts. The table includes compounds from WF4 fungi fermented on both rice and finger millet. [file Table5.DOCX]

**Supplemental Table S5.** NMR data for atomic assignments of compounds isolated from WF4 fungal extracts. The table includes compounds from WF4 fungi fermented on both rice and finger millet.

| **Atom No.** | **Carbon** | **^1^H-NMR** | **^13^C-NMR** |
| --- | --- | --- | --- |
| Compound 1 | | | |
|  |  |  |  |
| 2 | C | ------- | 158.3 |
| 3 | C | ------- | 157.2 |
| 4 | C | ------- | 142.2 |
| 5 | C | ------ | 134.9 |
| 6 | CH | 7.34 | 129.3 |
| 7 | CH | 7.32 | 126.4 |
| 8 | CH | 7.29 | 124.4 |
| 9 | CH | 7.09 | 122.1 |
| 10 | C | ------- | 133.1 |
| 3-OH |  | 12.2 |  |
| NH |  | 9.1 |  |
| Phenyl moiety |  |  |  |
| 1’ | C | ------ | 124.06 |
| 2’ | CH | 7.07 | 120.3 |
| 3’ | C | ------ | 120.9 |
| 4’ | CH | 6.82 | 116.6 |
| 5’ | CH | 6.72 | 115.2 |
| 6’ | CH | 6.71 | 114.6 |
| Ar-OH |  | 9.5 |  |
| Compound 2 | | | |
| 2 | C |  | 178.9 |
| 3 | C |  | 105.6 |
| 4 | C |  | 198.8 |
| 5 | CH | 3.7 | 67.20 |
| 6 | C |  | 195.01 |
| 7 | CH3 | 2.4 | 24.26 |
| 8 | CH | 1.89 | 38.3 |
| 9 | CH2 | 1.34 | 27.06 |
| 10 | CH3 | 0.95 | 12.42 |
| 11 | CH3 | 1.01 | 16.65 |
| Compound 3 | | | |
| 1 | C |  | 139.8 |
| 2 | C |  | 99 |
| 3 | C |  | 166.2 |
| 4 | CH | 7.3 | 101.9 |
| 5 | C |  | 166.8 |
| 6 | CH | 6.4 | 104.3 |
| 7 | C |  | 167 |
| CH3 |  | 2.8 | 25.8 |
| 1’ | C |  | 109 |
| 2’ | C |  | 154.4 |
| 3’ | CH | 6.6 | 100.6 |
| 4’ | C |  | 159.8 |
| 5’ | CH | 6.4 | 118.56 |
| 6’ | C |  | 140 |
| Compound 4 | | | |
| 1 | C |  | 138.2 |
| 2 | C |  | 98.9 |
| 3 | C |  | 165.1 |
| 4 | CH | 7.3 | 99.6 |
| 5 | C |  | 166.6 |
| 6 | CH | 6.59 | 102 |
| 7 | C |  | 166.6 |
| CH3 |  | 2.8 | 25.5 |
| OCH3 |  | 3.9 | 56.3 |
| 1’ | C |  | 109 |
| 2’ | C |  | 153 |
| 3’ | CH | 6.7 | 103.8 |
| 4’ | C |  | 164.6 |
| 5’ | CH | 6.6 | 118.1 |
| 6’ | C |  | 138.8 |
