# Supplementary material for: An endophytic fungus isolated from finger millet (Eleusine coracana) produces anti-fungal natural products
Source: Front Microbiol. 2015 Oct 21;6:1157. doi: 10.3389/fmicb.2015.01157 (PMC4612689; doi:10.3389/fmicb.2015.01157)
Supplement: Supplementary file 9 [file DataSheet1.DOCX]

**Supplementary methods**

**Toxicity assays**

For all toxicity assays described below, the original WF4 endophyte extract, which was dissolved in methanol, was replaced with water: 100 ml of the methanol extract was dried, and the residue was dissolved in 100 ml H_2_0 (7 mg/ml).

*Effect of WF4* *endophyte extracts on leaf health* – To generate the leaf materials for the toxicity assay, maize plants (hybrid 35F40) were grown in the Crop Science Greenhouse Facility (University of Guelph) using the following conditions: 16 h light (~600 µmol m^-2^ s^-1^) at 28˚C, 8 h dark at 23˚C, and 50% relative humidity. Plants were grown semi-hydroponically in pots containing Turface® clay, and irrigated with a nutrient solution containing: 0.4 g/L 28-14-14 fertilizer [28% total N (1.6% nitrate, 0.4% ammonium, 26% urea), 14% P_2_O_5_, 14% K_2_O], 0.4 g/L 15-15-30 fertilizer [15% total N (nitrate 8.8%, ammonium 2.95%, urea 3.25%), 15% P_2_O_5_, 30% K_2_O], 0.2 g/L NH_4_NO_3_, 0.4 g/L of MgSO_4_•7H_2_O and 0.03 g/L of micronutrient mix (S, Co, Cu, Fe, Mn, Mo and Zn). Leaf punches were taken from leaf blades at the six-week stage. Punches (1/4 inch diameter) were taken from young leaves. To conduct the toxicity assay, 24-well plates (Costar #3526, Fisher Scientific, USA) were filled with either deionized water containing 0.05 % Triton X-100 (negative control) or endophyte extract with 0.05 % Triton X-100 (2.5 ml per well). In each well was placed one young leaf punch (0.6 mm, punctured by cushioned grip hand punch, # 923095, Fiskars Brands, Inc, China), placed with either the adaxial or abaxial surface facing up. The experiment was repeated using five aqueous dilutions of WF4 extract (100%, 80%, 50%, 20%, and 10%). The multi-well plates were kept in the dark at room temperature. Pictures were taken of leaves after 2, 4 and 6 days of incubation, and the percentage surface area that showed lesions was quantified using Assess Software (Version 2.0, American Phytopathological Society) in comparison to controls.

*Effect of WF4* *endophyte extracts on seed germination -* Two types of seeds were used including maize (hybrid 35F40, Ridgetown College, Canada) and spring wheat (cultivar Quantum, Ridgetown College, Canada). Whatman filter papers were soaked in WF4 extract or water control. For each toxicity assay, three water-soaked filter papers and three extract-soaked filter papers (3 replicates) were distributed into six Petri dishes (100 mm x 15 mm). Into each Petri dish, ten seeds were added which had been surface-sterilized as described earlier. The experiment was repeated using five dilutions of WF4 extract (100%, 80%, 50%, 20%, and 10%). The Petri dishes were kept in the dark for 1-2 weeks, and the percentage of germination (hypocotyl emergence) was compared to the respective control.

*Effect of WF4 endophyte extracts on development of fruit flies (Drosophila melanogaster) –* Sokolowski Lab Fly Food was first prepared by mixing two sterilized solutions together: the first solution contained 25 g yeast extract dissolved in 100 ml of distilled water, and the second solution was composed of 50 g sucrose, 14.5 g agar, 0.5 g potassium phosphate, 4 g potassium sodium tartrate, 0.25 g sodium chloride, 0.25 g calcium chloride, 0.25 g magnesium chloride and 0.25 g iron (III) sulphate dissolved in 400 ml distilled water. After thoroughly mixing, 2.5 ml of propionic acid was added. The effect of WF4 endophyte extract on fruit fly egg development was tested by mixing 500 µl of the extract (or water, as control) into 6 ml of Sokolowski Lab Fly Food media; 1 ml of the resulting solution was pipetted into each well of a 12-well plate (1 ml volume/well). Into each well was added a 30 µl suspension of fruit fly eggs (in PBS, phosphate-buffered saline solution). A dissection stereomicroscope (Stemi 2000, Zeiss, Germany) was used to determine how many eggs were in each well, which was then adjusted to 30 eggs/well. The top of the 12-well plate was sealed with packing tape in which 4-5 holes were made using a syringe with a 20-22 gauge needle. The plates were incubated at 28˚C. Over a period of 23 days, fruit fly development was observed from larvae to pupae to adults. There were six wells used for the extract and the control.
